# A Fresh Look at Celery Collenchyma and Parenchyma Cell Walls Through a Combination of Biochemical, Histochemical, and Transcriptomic Analyses

**DOI:** 10.3390/ijms26020738

**Published:** 2025-01-16

**Authors:** Natalia Mokshina, Olga Sautkina, Oleg Gorshkov, Polina Mikshina

**Affiliations:** Kazan Institute of Biochemistry and Biophysics, FRC Kazan Scientific Center of RAS, Lobachevsky Str., 2/31, 420111 Kazan, Russia; sautkina@kibb.knc.ru (O.S.); o_gorshkov@mail.ru (O.G.); p.mikshina@gmail.com (P.M.)

**Keywords:** celery (*Apium graveolens*), growing petioles, mature petioles, collenchyma, parenchyma, vascular bundles, primary cell wall, RNA-Seq, polysaccharides

## Abstract

Celery (*Apium graveolens*) can be considered as a model plant for studying pectin-enriched primary cell walls. In addition to parenchyma cells with xyloglucan-deficient walls, celery petioles contain collenchyma, a mechanical tissue with thickened cell walls of similar composition. This study presents a comprehensive analysis of these tissues at both early and late developmental stages, integrating data on polysaccharide yield, composition, localization, and transcriptome analysis. Our results reveal that young collenchyma walls possess distinct polysaccharide compositions, including higher levels of rhamnogalacturonan I (RG-I), branched galactans, esterified homogalacturonan, and xyloglucan, compared to parenchyma cells. A significant number of genes encoding proteins involved in pectin methylesterification and acetylation were upregulated in young collenchyma. Different gene isoforms encoding glycosyltransferases involved in RG-I biosynthesis were activated in both collenchyma and parenchyma, suggesting potential variations in RG-I structure and function across different primary cell walls. We identified a set of potential glycosyltransferases involved in RG-I biosynthesis in collenchyma and proposed synthase complexes for heteromannan and heteroxylan. The transcriptome data not only confirmed known biochemical traits of celery cell walls but also provided deeper insights into the peculiarities of cell wall polysaccharide metabolism, thereby helping to narrow down candidate genes for further molecular genetic studies.

## 1. Introduction

The cell wall is a fundamental structure that distinguishes plant cells from animal cells and plays a crucial role in the growth, development, and function of plant cells, tissues, organs, and the entire plant organism. Primary cell walls are typically thin and extensible. In certain tissues, such as sclerenchyma and water-conducting elements, once cells reach their final size, they begin to develop secondary cell wall thickenings. However, collenchyma cells exhibit a different pattern: They grow with locally thickened primary cell walls [1,2,3]. The key hemicellulose in typical type I primary cell walls is xyloglucan, with minor amounts of heteromannans (HM) and heteroxylans (HX). Pectins in these walls are represented by homogalacturonan (HG) and rhamnogalacturonan I (RG-I), the latter of which bears galactan and arabinan side chains [4,5].

The collenchyma cell wall is polylamellar, displaying varying orientations of cellulose microfibrils across different layers. Once the cells achieve their final size, no further deposition of new wall material occurs in collenchyma [3]. Consequently, the processes related to both cell wall formation and growth are likely to be more pronounced in collenchyma compared to other cells with primary walls, due to the presence of an extensible, thickened cell wall. Biochemical analyses indicate that the composition of the collenchyma cell wall is generally similar to that of primary cell walls [2,6]. The typical primary cell walls of eudicots and non-commelinoid monocots consist of xyloglucan–cellulose networks embedded within a pectin matrix [5,7]. However, celery collenchyma cell walls are atypical; they contain a lower amount of xyloglucan and a higher proportion of HG [3]. Despite the lower xyloglucan content in collenchyma, it remains higher than that found in celery parenchyma cell walls [6,8]. Celery parenchyma cell walls are reported to contain minimal xyloglucan (2%) and approximately 50% pectins [8].

Enzymes involved in polysaccharide metabolism can be categorized into five key groups: (1) glycosyltransferases (GTs), which synthesize polysaccharides and are located in the plasmalemma (such as cellulose synthases, CESAs) or in the Golgi membrane; (2) methyl- and acetyltransferases, which add methyl and acetyl groups to polysaccharides, respectively, and are also located in the Golgi; (3) glycoside hydrolases (GHs) and lyases, which trim polysaccharides after their deposition into the cell wall; (4) cell wall hydrolases that act as transglycosylases, rearranging polysaccharide chains to form new interactions between xyloglucans and other polysaccharides (xyloglucan endo-transglucosylases/hydrolases (XTHs) belonging to GH16); and (5) esterases, which are localized in the cell wall and remove modifying groups (methyl or acetyl) from substituted polysaccharides [9,10,11]. Most of these plant protein families are organized and classified in the CAZy database [9]. In addition to these enzymes, there are also cell wall proteins that, although not enzymatically active, play crucial roles in cell wall organization. For example, expansins facilitate cell wall loosening by interacting with cellulose, thereby influencing cell growth and expansion [12].

Although methylesterification and acetylation are well-known modifications of plant polysaccharides, their effects on polysaccharide structure and properties are not fully understood nor are the molecular genetic aspects of these modifications. Pectin methylesterases (PMEs) catalyze the demethylesterification of pectins either in a block-wise or random manner. Block-wise demethylesterification typically results in cell wall stiffening due to calcium cross-linking between galacturonic residues, forming an egg-box structure. In contrast, random demethylesterification leads to cell wall loosening, as pectin becomes a substrate for polygalacturonases and pectate lyases [13,14]. PMEs can be classified into two types: Type-1, which possesses both the PME domain (PF01095) and a prodomain (PF04043) similar to the pectin methylesterase inhibitor (PMEI) domain, and Type-2, which lacks the prodomain [15]. The enzymatic activity of PMEs is regulated by PMEIs, which inhibit PMEs through direct binding to their catalytic domain. As a result, the level of HG methylesterification is controlled by the interplay between PME and PMEI activities [15]. Regarding methylesterification, it was only recently that Golgi-localized putative S-adenosyl methionine (SAM) transporters, essential for polysaccharide methylesterification, were discovered [16]. In plant cell wall polysaccharides, various residues can be methylesterified (GlcA of xylans, various monosaccharides in RG-II), but HG is the most abundantly methylesterified polysaccharide. The first candidate pectin methyltransferase (PMT) to be biochemically characterized in vitro was QUASIMODO2 (QUA2/TSD2), which methylesterifies HG using SAM as the donor substrate [17].

*O*-acetylation is a common modification found in nearly all cell wall polymers, with the exceptions of cellulose, mixed-linked glucan in grasses, and glycoproteins. This modification is mediated by reduced wall acetylation (RWA), which are xylan acetyltransferases, and trichome birefringence-like (TBL) proteins, which have various substrates [18]. While the biological function of *O*-acetylation in plant development and morphology is not fully understood, it is clear that it influences the rheological properties of cell wall polysaccharides and their interactions [18]. Pectin acetylesterases (PAEs) and certain GDSL lipases/esterases are known to be involved in polysaccharide deacetylation [19,20]. However, significant gaps remain in our understanding of the mechanisms of acetylation, its regulation, and its impact on plant physiology.

This study builds upon previous research on transcriptome profiling of celery petiole tissues [21], which, for the first time, explored the peculiarities of collenchyma cell wall formation from a gene expression perspective. The significant role of XTHs in the early stages of collenchyma development was highlighted. In the current paper, we investigate the dynamics of polysaccharide composition and content in parenchyma and collenchyma cell walls at two developmental stages (young and mature), along with a comprehensive analysis of the expression of genes encoding a wide range of cell wall-related proteins. We place particular emphasis on the biosynthesis of pectins and modifications of polysaccharides, such as methylesterification and *O*-acetylation. The RNA-Seq data for the young stage were supplemented with data for the mature stage and renormalized. Improvements were made to the RNA-Seq data assembly, correcting inaccuracies and enabling the identification of a greater number of expressed genes. The main objective of the current study is to link transcriptome data and biochemical analysis and to identify specific isoforms among multiple gene isoforms, whose products may contribute to the formation and modification of polysaccharide substrates. This study aims to enhance the use of celery as a model plant for investigating the roles of polysaccharides in plant cell walls and to establish a foundation for future reverse genetics research focused on plant cell wall biology, particularly in relation to celery. According to our findings, in addition to enhancing the expression of XTHs in collenchyma during the early stage of development, an observation also demonstrated in a previous study, we identified genes encoding the pectin biosynthetic enzymes. The expression of most of these genes was specifically increased in young collenchyma. RG-I in collenchyma cell walls contains branched galactan side chains. Coexpression analysis revealed pools of genes specifically expressed in different celery petiole tissues, encoding pectin-modifying enzymes, such as PMT, RWA, TBL, PME, PAE, and others. Additionally, sets of enzymes potentially involved in the biosynthesis and modifications of heteroxylans and heteromannans were established.

## 2. Results

### 2.1. Morphological Characterization of Celery Tissues at the Two Stages of Development

**Figure 1:**
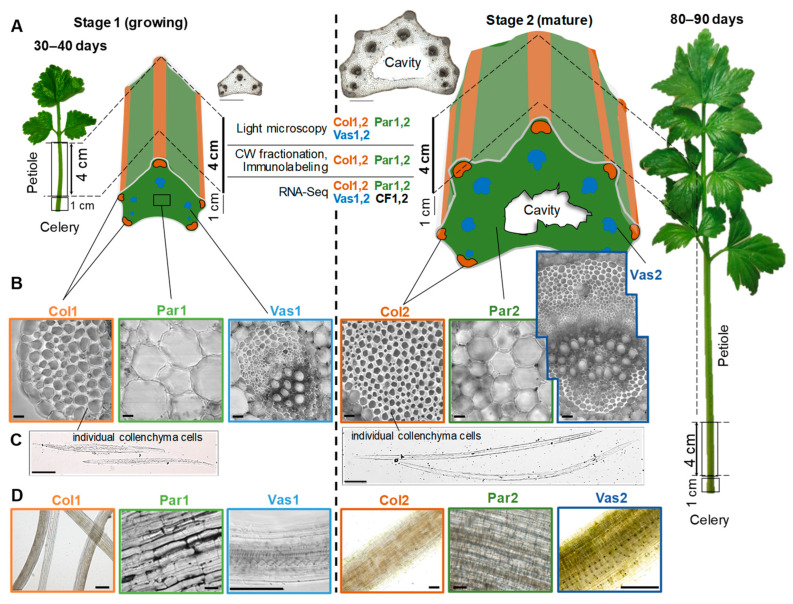
Scheme of sample collection, description, and types of experiments. (**A**) Scheme of sampling from the celery petiole at the two developmental stages, along with a list of samples and the types of analyses performed. Stage 1—growing stage; Stage 2—mature stage. Images of petiole cross-sections are also provided for both developmental stages. (**B**) Cross-sections of celery petiole fragments at the two developmental stages. (**C**) Isolated collenchyma cells from both a growing petiole and a fully matured petiole. (**D**) Isolated celery petiole tissues at the two developmental stages. Scale bars are as follows: (**A**)—1 mm, (**B**)—20 µm, and (**C**,**D**)—100 µm. CW—cell wall; Par—parenchyma; Col—collenchyma; Vas—vascular bundles; CF—collenchyma-free (petiole tissues excepted collenchyma).

To enhance understanding of the biological processes and tissue sampling strategy employed in this study, we present the morphological and anatomical characteristics of celery petiole tissues. In a single celery petiole, there are strands with varying numbers of cells: those formed at an early stage (with fewer cells), and those at the mature stage (with a greater number of cells [1], Figure 1A,B). The lengths of the growing petioles ranged from 5 to 6 cm, whereas mature petioles measured between 15 and 25 cm. Cross-sectional image analysis revealed that at the growing (young) stage of plant development, petioles from the periphery of the leaf rosette contained 3–5 collenchyma strands. In contrast, mature plants predominantly had petioles sampled from the center of the rosette, which featured 7 to 9 collenchyma strands and an equal number of vascular bundles. Throughout its development, the collenchyma comprised approximately 3–4% of the petiole area in transverse sections.

The collenchyma cells in young petioles had an elongated shape with tapering ends, and the number of individual collenchyma cells in a single strand, as observed in cross-sections, ranged from 15 to 330, depending on the stage of bundle development. In mature petioles, the collenchyma cells were fusiform and fiber-like, with the number of cells per strand varying from 1800 to 3800. According to a growth experiment conducted by [2], young petioles elongate along their entire length; however, elongation later becomes increasingly confined to the upper regions until it eventually ceases. The lengths of growing collenchyma cells ranged from 0.6 to 0.8 mm, while the average length of mature collenchyma cells was 1.1–1.2 mm, with some cells reaching up to 2 mm in length (Figure 1C).

The parenchyma cells in the growing petiole exhibited wavy outlines in transverse sections, while the cells in the mature petiole were more rounded. At a later stage of development, a hollow pith cavity formed (Figure 1A). The vascular bundle consists of a mixture of tissues, including collenchyma, phloem, procambium, protoxylem, and metaxylem vessels (Figure 1B,D). Essential oil channels may also be included.

### 2.2. Polysaccharide Composition of Celery Collenchyma and Parenchyma Cell Walls at Two Developmental Stages

Two fractions of polysaccharides were extracted from the washed cell walls of celery collenchyma and parenchyma isolated at the petiole elongation stage (young stage) and at the stage when the petiole had completed its growth (mature stage). Sequential extraction was performed using a chelating agent and concentrated alkali. The first fraction was rich in pectins, while the second predominantly consisted of cross-linking glycans (hemicelluloses) [22].

**Figure 2:**
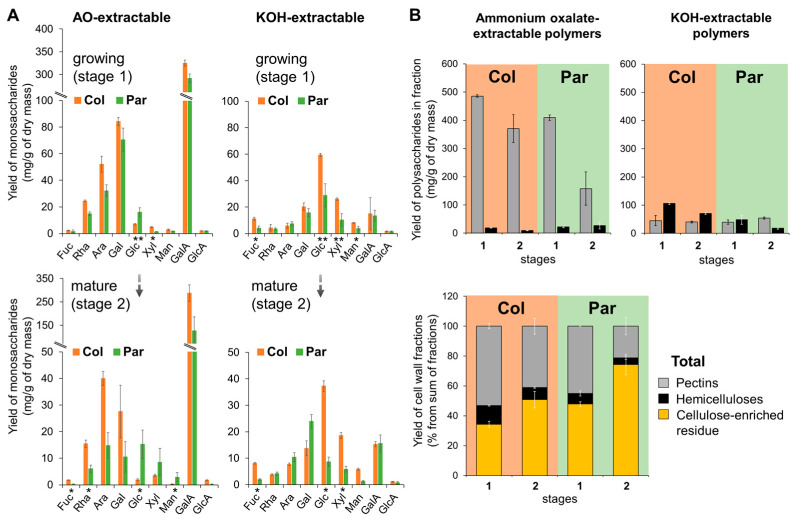
(**A**) Monosaccharide composition of cell wall fractions (mg/g of cell wall dry mass) from celery collenchyma (Col) and parenchyma (Par) at two developmental stages (Stage 1—growing stage, Stage 2—mature stage). * indicates a significant difference at *p* < 0.05, and ** indicates a significant difference at *p* < 0.1. AO-extractable refers to the pectin-enriched fraction; KOH-extractable refers to the hemicellulose-enriched fraction. (**B**) Yield of different groups of polysaccharides (mg/g of cell wall dry mass) from collenchyma and parenchyma cell wall fractions. The relative content of cell wall fractions was calculated by summing the portions of pectins and hemicelluloses obtained from AO- and KOH extractions, while the remaining portion of the cell wall was referred to as the cellulose-enriched residue. Error bars represent the standard deviations.

Based on the monosaccharide analysis of cell wall fractions, we concluded that AO-extractable polymers (pectins) mainly consist of HG and RG-I, probably, with arabinan and galactan side chains, showing no significant differences between collenchyma and parenchyma cell walls at the early developmental stage (Figure 2A). Both arabinose and galactose were present in all analyzed fractions, with slightly higher concentrations in collenchyma compared to parenchyma in the AO-extractable fraction. At the mature stage, the level of HG in collenchyma cell walls remained unchanged, while it decreased significantly in parenchyma cell walls (Figure 2A).

Notable differences in KOH-extractable polymers were observed for xylose, fucose, and glucose, with these monosaccharides being more abundant in collenchyma cell walls. These monosaccharides are components of xyloglucan, and the differences became more pronounced at maturity, despite an overall decrease in monosaccharide content.

Both primary cell walls were enriched with pectins, comprising approximately 57% in collenchyma cell walls and 50% in parenchyma cell walls at the early stage. As the cells matured, the pectin content decreased in both tissues, with a more pronounced reduction observed in parenchyma (Figure 2B). In the cell walls of growing collenchyma, hemicelluloses accounted for about 12%, while in the parenchyma, they comprised about 8% (sum of all fractions). In mature cell walls, the hemicellulose content further decreased in both tissues. Due to the lower pectin and hemicellulose content, parenchyma cell walls exhibited a higher proportion of cellulose (Figure 2B). Some portion of pectins and hemicelluloses remained in the cellulose-enriched residues from both tissues and was included in the calculation of the total polysaccharide composition of cell walls (Figure 2B).

### 2.3. Distribution of Pectin Polysaccharide Epitopes in Collenchyma and Parenchyma Cells at Two Developmental Stages

We employed a wide panel of antibodies to investigate the distribution of polysaccharide epitopes in celery collenchyma and parenchyma cells at two distinct developmental stages. The key characteristics of the antibodies used are detailed in Table S1.

**Figure 3:**
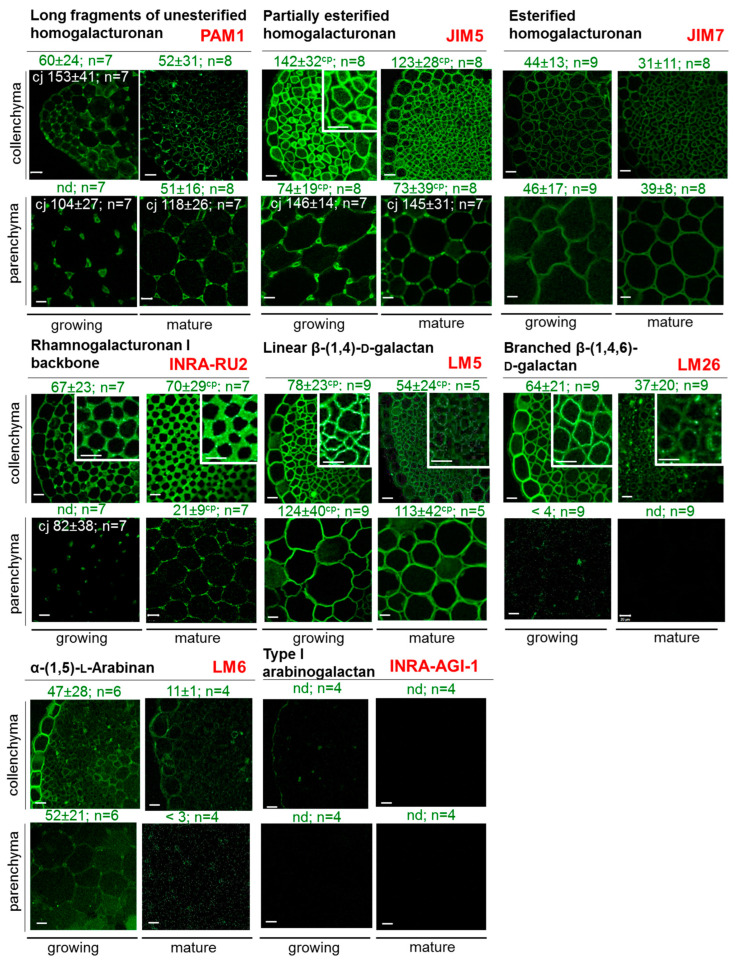
Immunolocalization of pectin polysaccharide epitopes in collenchyma and parenchyma cell walls at two developmental stages. In some images, the zoomed area is indicated by a white frame. Fluorescence quantification was performed using ImageJ software, with *n* representing the number of analyzed cross-sections. cj—Fluorescence in cell junctions; nd—fluorescence not detected; ^cp^ indicates significant differences between collenchyma and parenchyma within the respective developmental stage (Student’s *t*-test, *p* < 0.05). Scale bar: 20 μm.

Regarding the main components of pectic substances, homogalacturonans (HGs), it was found that unesterified HG (PAM1; [23,24]) in the collenchyma cell wall was predominantly detected in the inner layer of the wall. In contrast, in parenchyma cells, long fragments of unesterified HG were primarily found at tricellular junctions. For collenchyma cell walls, labeling of partially esterified HG (JIM5; [24]) was detected along the entire cell perimeter, exhibiting greater intensity at the early developmental stage; however, this intensity sharply declined in mature collenchyma cell walls (Figure 3). In parenchyma cells, HG with a low degree of esterification was detected in two distinct regions: at the interfaces between adjacent cells, where labeling was more pronounced, and within the cell walls themselves, where the labeling intensity was lower (Figure 3). Notably, this labeling pattern remained consistent at the mature stage. The labeling of esterified HG (JIM7; [24]) was uniformly distributed in the inner regions of the collenchyma cell wall and across all surfaces of the parenchyma cell walls. No significant differences in labeling were observed between the two tissue types or across the various developmental stages.

Using INRA-RU2 antibodies, which are specific for the RG-I backbone [25], we clearly demonstrated the accumulation of RG-I in collenchyma cell walls, with levels increasing at the later developmental stage. In parenchyma, the RG-I backbone was primarily detected in cell corners, but in mature parenchyma, the labeling extended across the entire cell surface (Figure 3). RG-I has galactan and arabinan side chains attached to its backbone [5,26]. Linear β-1,4-galactans (LM5; [27]) were detected in the inner part of the collenchyma cell wall as well as throughout the parenchyma cell wall at both developmental stages. Labeling was more intense in parenchyma cell walls (Figure 3), whereas in collenchyma cell walls, labeling diminished at the mature stage. Despite the lack of significant differences in galactose content between collenchyma and parenchyma cell walls, (1→6)-branched β-(1→4)-d-galactans were specifically identified in the inner part of collenchyma cell walls, predominantly at the early stage (LM26; [28]). The labeling of linear α-1,5-arabinans and type I arabinogalactans (LM6; [29,30]; INRA-AGI-1; [31]) was weak in both tissues at both developmental stages. Although biochemical analysis detected arabinose in all cell wall fractions, it is possible that epitope masking by other cell wall polymers may have occurred, affecting the visibility of these components.

### 2.4. Distribution of Hemicellulose and Crystalline Cellulose Epitopes in Collenchyma and Parenchyma Cell Walls at Two Developmental Stages

**Figure 4:**
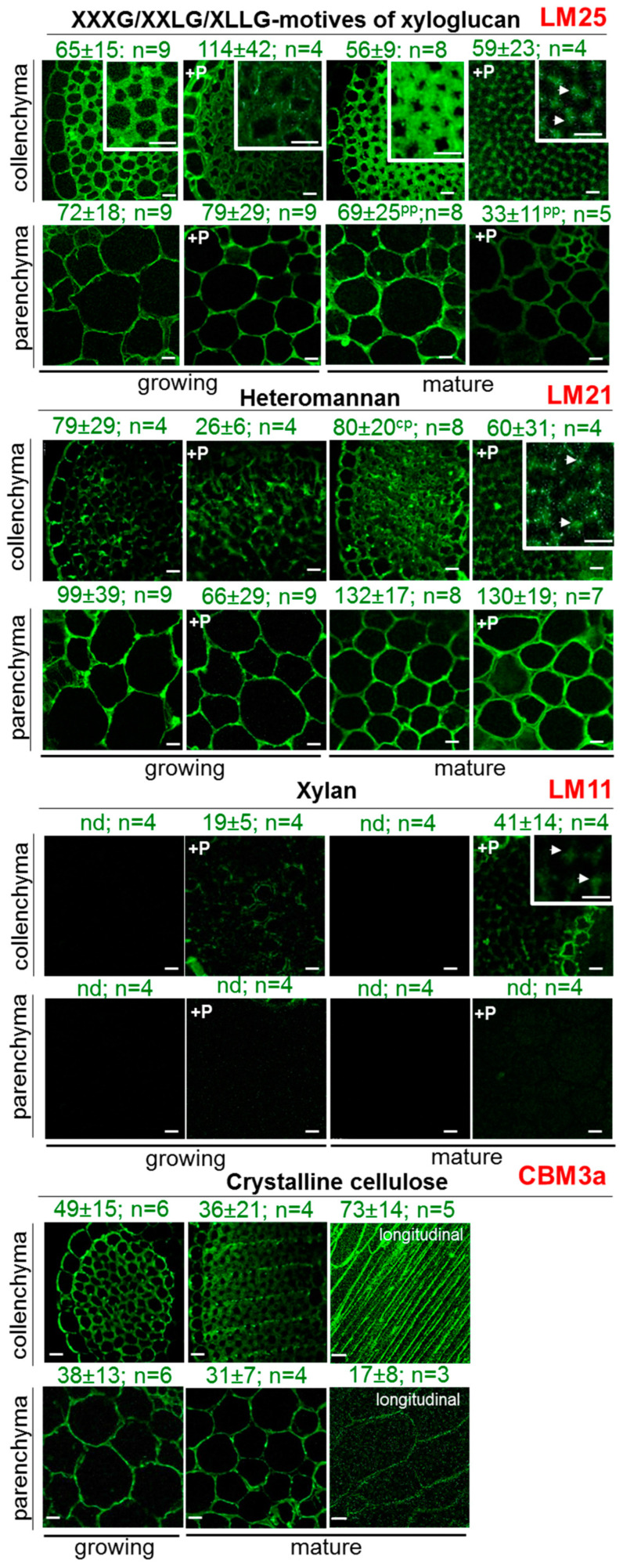
Immunolocalization of xyloglucan (LM25), mannans (LM21), xylan (LM11), and crystalline cellulose (CBM3a) epitopes in collenchyma and parenchyma cell walls at two developmental stages. +P indicates treatment with pectinase to remove masking effects. nd—Fluorescence not detected; ^cp^ and ^pp^ indicate significant differences between collenchyma and parenchyma within the developmental stage and between parenchyma samples before and after pectinase treatment, respectively (Student’s *t*-test, *p* < 0.05). White arrows highlight the outer regions of the cell wall near the junctions. Scale bar: 20 μm.

The hemicellulose content in the primary cell walls of both collenchyma and parenchyma was found to be 5–6 times lower than that of pectins (Figure 2; [8,21]). Epitopes for xyloglucan were distributed uniformly across the entire surface of cell walls in both tissues (LM25; [32]), showing no significant differences between them (Figure 4). Notably, during maturation, the intensity of xyloglucan labeling remained stable. Treatment with pectinase to remove pectins that may mask some portion of xyloglucan epitopes resulted in a reduction in the fluorescence intensity of xyloglucan labeling in the cross-sections at both stages. However, at the mature stage, the removal of pectins led to the labeling of xyloglucan in the outer regions of the cell wall near the cell junctions.

Despite the relatively low and similar content of mannans in both tissues, their labeling (LM21; [33]) was much more intense in the parenchyma cell walls than in the collenchyma. This observation suggests that mannans are more accessible to antibodies in parenchyma. As maturation progressed, the content of mannans increased in both tissues; however, in collenchyma, the labeling remained less distinct. Treatment with pectinase did not affect the labeling of mannans in the parenchyma cell walls. In contrast, in collenchyma, mannans were primarily localized in the outer regions of the cell wall near the cell junctions, and fluorescence intensity decreased compared to untreated sections.

Xylan labeling (LM11; [34]) was not detected in either tissue at the early developmental stage. However, faint fluorescence was observed in collenchyma cell walls after potential masking was removed. In mature cell walls, weak xylan labeling was noted in the outer regions of the cell wall near the cell junctions and on the periphery of the collenchyma strand following pectinase treatment (Figure 4). A similar pattern of xylan labeling has been previously described [3]. Interestingly, in contrast to our findings, which indicated that xylan was present in small amounts in the collenchyma cell walls at early stages, a previous study reported that the LM11 epitope was undetectable in the collenchyma walls of young celery plants, even after pectate lyase pre-treatment, and weak labeling of the LM11 epitope was observed in collenchyma walls at the mature stage after pectate lyase pre-treatment [3].

Xylan labeling (LM11; [34]) was not detected in either tissue at the early developmental stage. However, faint fluorescence was observed in collenchyma cell walls after potential masking was removed. In mature cell walls, weak xylan labeling was noted in the outer regions of the cell wall near the junctions and on the periphery of the collenchyma strand following pectinase treatment (Figure 4). A similar pattern of xylan labeling has been previously described [3]. Interestingly, in contrast to our findings, which indicated that xylan was present in small amounts in the collenchyma cell walls at early stages, the LM11 epitope was undetectable in the collenchyma walls of young celery plants, even after pectate lyase pre-treatment. However, at later stages, weak labeling of the LM11 epitope was observed in collenchyma walls following pectate lyase pre-treatment [3].

Collenchyma cell walls exhibited more intensive binding with CBM3a, which targets crystalline cellulose [35], compared to parenchyma cell walls (Figure 4). This binding was particularly pronounced in longitudinal sections.

### 2.5. Transcriptome Analysis of Celery Tissues at Two Developmental Stages

**Figure 5:**
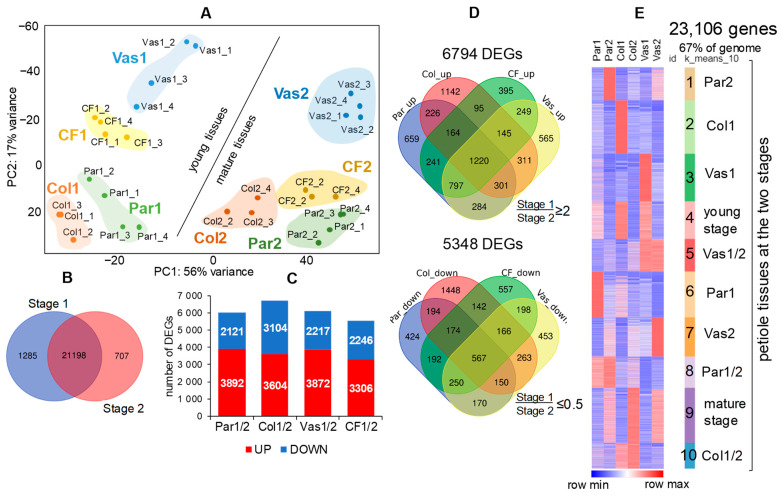
(**A**) Principal component analysis (PCA) of RNA-Seq data for celery petiole tissues at the two developmental stages (1—young tissues, 2—mature stages). (**B**) Venn diagram showing the genes expressed in celery petiole at early (1) and mature (2) stages (TGR ≥ 16 at least in one sample). (**C**) Number of up- and downregulated genes in tissues across the two developmental stages (FC ≥ |2|, padj ≤ 0.01). (**D**) Venn diagram of differentially expressed genes (DEGs), up- and downregulated (FC ≥ |2|, padj ≤ 0.01) at the two developmental stages, for each tissue. (**E**) Clustering of 23,106 expressed celery genes (k-means, Pearson correlation, 10 clusters, TGR ≥ 16 at least in one analyzed sample, https://software.broadinstitute.org/morpheus/, accessed on 20 August 2024). A relative color scheme uses the minimum and maximum values in each row to convert values to colors. The data represent the TGR of expressed genes in each cluster. Par—parenchyma; Col—collenchyma; Vas—vascular bundles; CF—collenchyma-free (petiole tissues excepted collenchyma).

We sequenced the transcriptomes of four samples: collenchyma, vascular bundle, parenchyma, and the collenchyma-free tissue samples representing part of the petiole without collenchyma strands at the two developmental stages. The RNA-Seq data are crucial for understanding the cell processes and the expression of genes related to cell wall biosynthesis and modifications. A total of approximately 680 million reads were obtained, with 95% achieving a quality score of Q30. Of these, 97% were successfully mapped to the reference genome of *Apium graveolens* (http://bio2db.com/download.html, accessed on 20 August 2024). The correlation between expression matrices of the samples, assessed through multidimensional scaling (principal component analysis, PCA), indicated strong convergence among biological replicates (average correlation coefficient *r* > 0.93 ± 0.023) (Figure 5A). The first two principal components accounted for over 70% of the variability between samples.

In the context of the transcriptome, collenchyma and parenchyma samples were found to be closer to each other at both developmental stages. This observation aligns with the understanding that collenchyma shares more morphological and physical characteristics with parenchyma tissues, leading some researchers to consider collenchyma as a type of thick-walled parenchyma [36]. CF (collenchyma-free) samples were positioned between Par (parenchyma) and Vas (vascular bundles) on the PCA plot, confirming the relevance of the sampling and transcriptomic data, as CF samples encompass both tissue types (Figure 5A). For clustering analysis, CF samples were excluded due to their representation of a mixture of tissues, but they were included in the coexpression analysis.

A total of 23,106 genes (67% of the genome) were expressed in celery petiole tissues (TGR ≥ 16 in at least one sample). Of these, 92% of expressed genes overlapped between the two developmental stages, with a higher number of specifically expressed genes (TGR ≥ 16) observed at the young stage (Figure 5B). As expected, young tissues exhibited a greater number of upregulated genes (Figure 5C,D). The highest number of differentially expressed genes (DEGs) in collenchyma samples indicates high transcriptional activity, likely driven by intense metabolic processes in this tissue.

**Figure 6:**
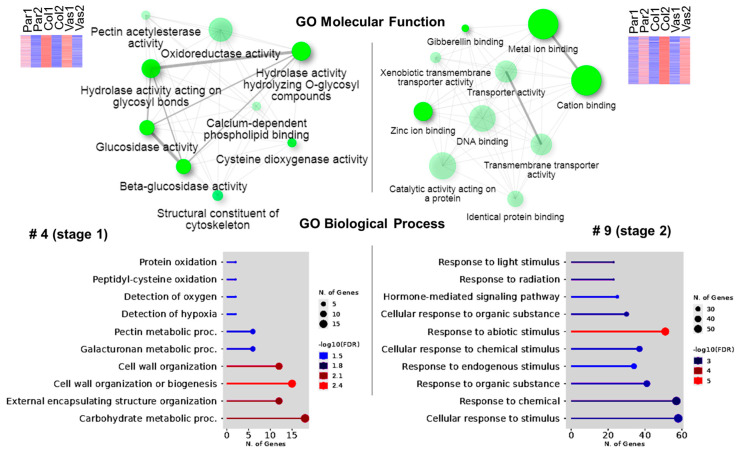
Gene Ontology (GO) categories (Molecular Function and Biological Process) for upregulated genes from clusters 4 and 9. The GO annotation was performed using Arabidopsis orthologous genes. Genes were filtered based on fold change in gene expression levels: For cluster 4, the criteria were Par1/Par2, Col1/Col2, and Vas1/Vas2 ≥ 4, while for cluster 9, the criteria were Par2/Par1, Col2/Col1, and Vas2/Vas1 ≥ 2. Par—parenchyma; Col—collenchyma; Vas—vascular bundles.

All expressed genes were clustered into 10 groups based on their expression dynamics (Figure 5E, Table S2). The gene sets in each cluster primarily correspond to specific cell types and tissues, as confirmed by GO analysis based on *Arabidopsis thaliana* orthologous genes (Table S3). For example, in mature parenchyma (cluster 1), processes associated with plant organ senescence were activated, particularly genes for proteins involved in the ethylene signaling pathway (Table S2). In young collenchyma (cluster 2), a significant portion of upregulated genes encoded cell wall-related proteins and proteins involved in lipid metabolism. Clusters 3 and 5, which included genes upregulated in vascular bundles, contained genes encoding proteins involved in cell cycle regulation and phloem or xylem histogenesis. Cluster 4, which combined genes upregulated in young tissues, mainly contained genes encoding cell wall-related proteins, with specific groups associated with pectin metabolism and glucosidase activity (GO Molecular Function; Figure 6). In mature tissues (cluster 9), genes-encoding proteins involved in responses to various stimuli were activated, with functions related to transport processes, gibberellin signaling, and the binding of metal ions, zinc ions, cations, and DNA (GO Molecular Function; Figure 6). These gene sets provide physiological relevance to the transcriptomic data obtained. Moreover, the validation of transcriptomic data using qPCR was conducted in both the current and previous research (Figure S1; [21]).

#### 2.5.1. Expression of Genes Encoding Cell Wall-Related Proteins in Celery Petiole Tissues

Given the focus on the unique features of celery collenchyma and parenchyma cell walls, we interpreted the transcriptomic data through the lens of cell wall metabolism. Specifically, we selected genes that encode proteins involved in the biosynthesis and modification of cell wall polysaccharides, including cellulose, xyloglucan, xylan, mannan, RG-I, and HG. This selection was based on homologous gene data from Arabidopsis and other plants available in the CAZy [9], UniProt [37], Phytozome [38], and TAIR [39] databases. Some protein groups, such as BURP-domain proteins and GDSL lipases/esterases, could not be directly linked to specific polysaccharides due to limited information available in the literature or databases. For instance, some BURP-domain proteins are known to interact with α-expansins [40], while certain GDSL lipases/esterases may exhibit deacetylase activity [20].

**Figure 7:**
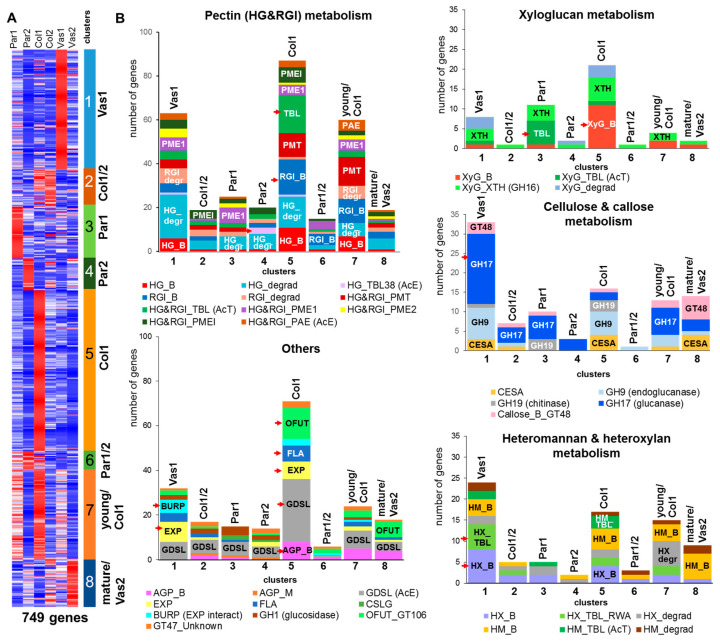
(**A**) Clustering of 749 expressed genes encoding cell wall-related proteins (k-means, Pearson correlation, 8 clusters, TGR ≥ 16 at least in one analyzed sample, https://software.broadinstitute.org/morpheus/, accessed on 20 August 2024). (**B**) Distribution of a set of genes encoding proteins involved in biosynthesis and modifications of crucial polysaccharides between clusters (see Table 1). B—biosynthesis; degr—degradation; XyG—xyloglucan; RG-I—rhamnogalacturonan I; HG—homogalacturonan; HM—heteromannan; HX—heteroxylan; PMT—pectin methyltransferase; TBL—trichome birefringence-like proteins (acetyltransferase); PME(I)—pectin methylesterase (inhibitor); PAE—pectin acetylesterase; AcT—acetyltransferases. The red arrow marks gene groups especially abundant in the cluster. A list of genes, their expression, and their distribution between cell wall-related groups and clusters are given in Table S4. Par—parenchyma; Col—collenchyma; Vas—vascular bundles.

In total, 749 genes related to various CAZy families and other cell wall-related proteins were expressed in celery petiole tissues. These genes were grouped into eight clusters (Figure 7A), with approximately 50% distributed between two clusters associated with upregulated genes in young collenchyma (cluster 5) and vascular bundles (cluster 1). The third largest cluster, cluster 7, contained genes that were upregulated in young tissues, particularly in young collenchyma, and exhibited a similar set of gene families and number of members as those in cluster 5. This similarity can be attributed to the comparable dynamics of gene expression in these clusters, both of which peak in collenchyma.

The genes in the leading clusters (1 and 5) highlight the distinct characteristics of two types of cell walls: secondary cell walls in vessels (vascular bundles) and primary cell walls in collenchyma. The expression of genes for different isoforms of cellulose synthases (CESAs) serves as reliable “markers” for these wall types. Genes for secondary cell wall-related CESAs (CESA4, 7, 8; [41]) were upregulated in vascular bundles, while genes encoding primary cell wall-related CESAs (CESA1, 3, 6; [42]) were upregulated in collenchyma (Table S4). In vascular bundles, genes for proteins involved in the biosynthesis of xylan, a major hemicellulose of secondary cell walls, were activated, along with numerous genes encoding proteins related to pectin modifications, such as polygalacturonases, pectate and rhamnogalacturonan lyases, methyl- and acetyltransferases, and esterases (cluster 1; Table S4). Although heteromannans and heteroxylans are considered minor components of the collenchyma cell wall [2], genes encoding their biosynthetic enzymes were specifically upregulated in collenchyma and, to a lesser extent, in parenchyma. A distinct set of genes for mannan-related biosynthetic enzymes was activated in vascular tissues (Figure 7B). Notably, 42% of all genes encoding β-1,3-glucanases (GH17), enzymes typically associated with plant–microbe interactions, were found in cluster 1. These enzymes may accumulate in vascular tissues to help prevent pathogen attacks and degrade callose during the development of plasmodesmata [43].

In young collenchyma, the biosynthesis of xyloglucan is significantly enhanced, as evidenced by the upregulation of genes encoding related proteins (Figure 7B). During this stage, extensive modifications of xyloglucan occur after its deposition into the cell wall, with approximately one-third of the xyloglucan endotransglucosylase/hydrolase (XTH) genes being highly expressed and activated ([21], Table S4).

As previously noted, there is a significant decrease in pectin content in the parenchyma cell walls at the mature stage. The transcriptomic analysis did not reveal a large number of gene sets encoding glycan-degrading enzymes activated in mature parenchyma cells. However, we observed a significant increase in the fold change in gene expression values for several genes, including 1,3-beta-glucosidase *Ag4G00118* (Par2/Par1 = 19, and Col2/Col1 = 22), polygalacturonase *Ag4G00772* (Par2/Par1 = 167), polygalacturonase *Ag6G00194* (Par2/Par1 = 48), β-galactosidase *Ag10G01453* (Par2/Par1 = 13), and 1,3-beta-glucosidase *Ag2G02119* (Col2/Col1 = 11).

#### 2.5.2. Pectin Biosynthesis

Given the activation of a large number of genes encoding products involved in the pectin biosynthesis and modification in young collenchyma, it is likely that these processes are prominently represented. Approximately 60% of these genes were distributed between clusters 5 and 7 (Figure 7B). Additionally, the increase in pectin content (specifically, an increase in galactose and arabinose) in collenchyma has been confirmed by biochemical analysis in this study. Some enzymes that utilize RG-I and/or HG as substrates, such as PME and PAE, were referred to as the “HG and RGI” group. Distinct sets of genes for proteins involved in HG biosynthesis and modification were upregulated in young vascular bundles (cluster 1), parenchyma (cluster 4), and collenchyma (cluster 5) (Figure 7B).

**Figure 8:**
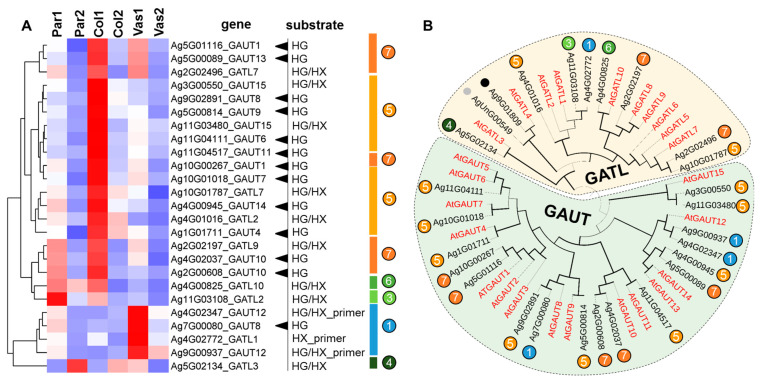
(**A**) Clustering of GAUT and GATL genes (PF01501) based on their expression levels (TGR values) in celery petiole tissues. (**B**) Phylogenetic dendrogram of Arabidopsis (red) and celery (black) GT8 family members (GAUTs and GATLs). The model of substitution used is Q.yeast+R5, with ultrafast bootstrap support set to 10,000. Branches with maximum bootstrap values are represented with maximum width. HG—homogalacturonan; HX—heteroxylan. Substrate specificity was assigned according to the literature [44,45,46,47] and description in UniProt [35]. Cluster numbers are given in circles, using the same color scheme as in Figure 7. These clusters include genes that are upregulated in designated tissues: cluster 1—Vas1; 3—Par1; 4—Par2; 5—Col1; 6—Par1/2; 7—young/Col1. Par—parenchyma; Col—collenchyma; Vas—vascular bundles. A gray circle denotes low expression levels (TGR < 16 in all samples), while a black circle indicates no expression.

We precisely assessed the expression levels of galacturonosyltransferase (*GAUT*) and *GAUT-like* (*GATL*) genes in celery petiole tissues (Figure 8). These genes encode members of the glycosyltransferase family 8 (GT8), which are involved in pectin and/or xylan biosynthesis. The homogalacturonan–galacturonosyltransferase (HG–GalAT) activity has previously been demonstrated for GAUTs 1, 4, 10, 11, 13, and 14, as well as for the GAUT1–GAUT7 complex [44]. GAUT7 interacts with GAUT1 to form GAUT1–GAUT7 HG–GalAT complex [45]. Additionally, it has been shown that in pollen tubes, GAUT5 and GAUT6, homologs of GAUT7, also target GAUT1 to the Golgi apparatus [46].

A significant portion of the *AgrGAUT* and *AgrGATL* genes (18 out of 25) was found to be upregulated in young collenchyma (cluster 5) as well as in other young tissues, with the highest expression observed in collenchyma (cluster 7) (Figure 8). In addition to the genes primarily upregulated mainly in collenchyma, several genes were activated in both parenchyma and collenchyma (*AgrGAUT10*, *AgrGATL2*, *9*, *10*). In young vascular bundles, *AgrGAUT12* and *AgrGATL1* were upregulated. The enzymes encoded by these genes are predicted to be galacturonosyltransferases, which play a role in pectin assembly and in synthesizing a complex glycan primer for xylan biosynthesis [47]. The vascular bundle comprises a mix of tissues, including those with primary cell walls enriched with pectins, while glucuronoarabinoxylan biosynthesis generally occurs in vessels and fibers with secondary cell walls. In mature tissues, the expression of most *AgrGAUTs* and *AgrGATLs* decreased. The expression levels of genes encoding proteins involved in pectin biosynthesis suggest a higher content of homogalacturonan in collenchyma. Notably, we observed an increased content of galacturonic acid in the collenchyma compared to the parenchyma at the mature stage (Figure 2A).

**Figure 9:**
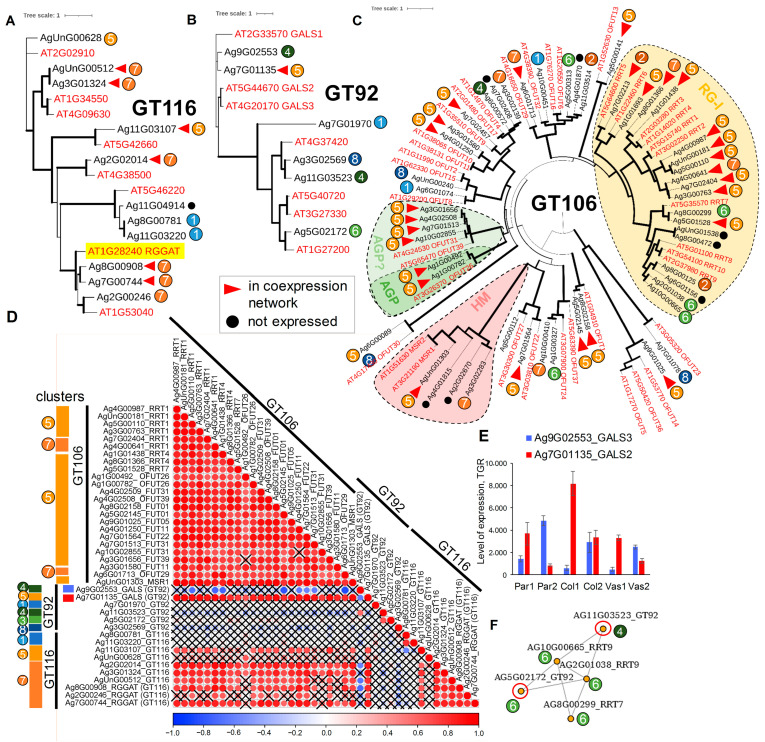
(**A**) Phylogenetic dendrogram of Arabidopsis (red) and celery (black) GT116 family members (PF04765). The model of substitution VT + I + G4, ultrafast bootstrap support 10,000. (**B**) Phylogenetic dendrogram of Arabidopsis (red) and celery (black) GT92 family members (PF13704). The model of substitution WAG + F + G4, ultrafast bootstrap support 10,000. (**C**) Phylogenetic dendrogram of Arabidopsis (red) and celery (black) GT106 family members (PF10250). The model of substitution WAG + F + I + R6, ultrafast bootstrap support 10,000. Branches with maximum bootstrap values have maximum width. HM—heteromannan; RG-I—rhamnogalacturonan I; AGP—arabinogalactan protein. Cluster numbers are given in circles using the same color scheme as in Figure 7. These clusters include genes that are upregulated in designated tissues: cluster 1—Vas1; 2—Col1/2; 3—Par1; 4—Par2; 5—Col1; 6—Par1/2; 7—young/Col1; 8—mature/Vas2. Par—parenchyma; Col—collenchyma; Vas—vascular bundles. Red triangles mark coexpressed genes. (**D**) Correlation coefficient plot based on RNA-Seq data for selected genes (GT106, GT92, and GT116) in celery tissues. Genes positively and negatively coexpressed with others are marked by red and blue dots, respectively. Dot size and color indicate the absolute value of Pearson’s correlation coefficient (*p* < 0.01; insignificant *p*-values are labeled with X, Table S5). (**E**) Expression of celery galactan synthases AgrGALS2 and AgrGALS3 in petiole tissues. (**F**) Coexpression of two members of GT92 (red circles) with some members of the RRT clade.

Most genes for proteins associated with RG-I biosynthesis were upregulated in young collenchyma (clusters 5 and partially 7). Key glycosyltransferases involved in RG-I biosynthesis include RG-I rhamnosyltransferases (RRTs, GT106 family; [48]) and RG-I galacturonosyltransferases (RG-I–GalATs, named as RGGATs, GT116 family; [49]) (Figure 9). A phylogenetic analysis of GT106 family members identified 18 genes, with 15 expressed in celery petiole tissues. This includes the RRT clade (RRT1-10) [48] and the mannan synthase-related (MSR) clade [50] (Figure 9C). Other related subgroups within GT106 comprise genes encoding glycosyltransferases involved in the biosynthesis of pectic type-II arabinogalactans (PAGR; [51]). The remaining genes belong to clades annotated as *O*-fucosyltransferases (OFUTs), although experimental evidence for their activity is rather scarce.

Interestingly, the celery *RRT* genes (orthologous to *AtRRT1-7*) were upregulated in young collenchyma and primarily belong to clusters 5 or 7. Two celery genes from cluster 6, *Ag2G01038* and *Ag10G00665* (both orthologous to *AtRRT9*), were upregulated in parenchyma, suggesting that distinct sets of RRTs may be involved in RG-I biosynthesis (Tables S2 and S4). The upregulation of several *AgrRRTs* in young collenchyma may indicate the necessity for a complex of different RRT isoforms for this process, a hypothesis that warrants further investigation. A correlation coefficient plot based on RNA-Seq data for all *GT106* members revealed that a significant number of these genes were activated in young collenchyma and exhibited high correlation coefficients (Figure S2, Table S5). A list of coexpressed *GT106* members, along with genes from the RRT clade, included those encoding MSRs, PAGRs, and some OFUTs. Genes with high correlation coefficients were further analyzed for coexpression with genes encoding galactan synthases (GT92; [52]) and RGGATs (GT116) (Figure 9).

Out of the six genes encoding GT92 members, only one (*Ag7G01135*, orthologous to the Arabidopsis galactan synthase *AtGALS2*) (Figure 9B) exhibited a high correlation coefficient with selected genes encoding GT106 members (Figure 9C,D). Meanwhile, 6 out of 10 *GT116* genes were found to co-express with *GT106* genes, including the *AgrRRTs* (Figure 9C). Notably, in Arabidopsis, galacturonosyltransferase activity has been confirmed only for AT1G28240 (RGGAT, GT116; [49]). The results from this study suggest that other GT116 members may also possess galacturonosyltransferase activity. We propose AT5G42660, AT4G38500, and AT1G34550 (along with their orthologs) as potential RGGATs. The activation of multiple RGGAT isoforms, similar to other backbone synthases such as RRTs, supports the hypothesis that RG-I synthases function as a complex rather than as individual glycosyltransferases, a theory that requires further investigation. The phylogenetic tree constructed for GT116 members of Arabidopsis and celery indicates that all potential RGGATs belong to different clades (Figure 9A). Two genes encoding GT116 members (*Ag8G00781* and *Ag11G03220*, both orthologs of *AT5G46220*) were upregulated in vascular tissues, although their expression levels were very low. *Ag9G02553*, an ortholog of *AtGALS3* (Figure 9B), was found to be negatively coexpressed with several genes encoding GT106 family members (Figure 9D). The expression of *AgrGALS3* was upregulated at later stages across all tissues, with the highest levels observed in parenchyma (Figure 9E). Interestingly, genes for two members of GT92, which belong to a distinct clade that includes galactan synthases, were found in a coexpression network with *AgrRRTs* (*AgrRRT7* and *AgrRRT9*). These *AgrRRTs* were not part of the previously described coexpression network of genes upregulated in collenchyma (clusters 5 and 7). For the enzymes encoded by these two genes, their activity has not been established experimentally; thus, we can only speculate that they may function as galactan synthases based on the coexpression of their genes with *RRTs*.

#### 2.5.3. Pectin Methylesterification

HG is the most abundantly methylesterified polysaccharide, with galacturonic acid (GalA) being the primary site of methylesterification [53]. Additionally, glucuronic acid (GlcA) residues in xylan, arabinogalactan proteins [54,55], and various residues in RG-II can also undergo methylesterification [56]. The degree of methylesterification of HG is a key factor affecting cell wall elongation [57].

**Figure 10:**
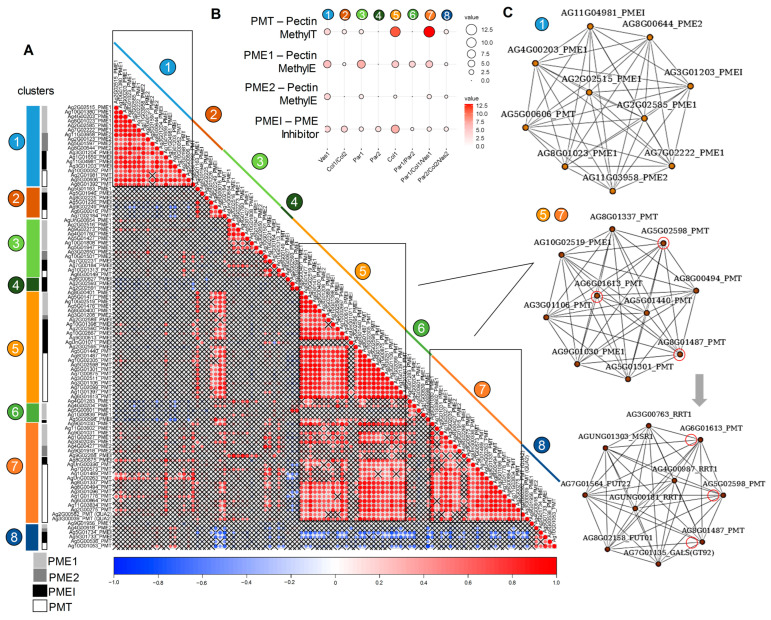
(**A**) Correlation of expression among genes encoding pectin methyltransferases (AgrPMTs), pectin methylesterases (AgrPME1 and AgrPME2), and PME inhibitors (AgrPMEI) in celery tissues, aligned with the clustering of genes (in circles) for cell wall-related proteins. Cluster numbers are given in circles using the same color scheme as in Figure 7. These clusters include genes that are upregulated in designated tissues: cluster 1—Vas1; 2—Col1/2; 3—Par1; 4—Par2; 5—Col1; 6—Par1/2; 7—young/Col1; 8—mature/Vas2. Par—parenchyma; Col—collenchyma; Vas—vascular bundles. Genes positively and negatively coexpressed with others are marked by red and blue dots, respectively. The size and color of the dots indicate the absolute value of Pearson’s correlation coefficient (*p* < 0.01; insignificant *p*-values are labeled with X, Table S5). Black frames designate large coexpressed gene groups. (**B**) Number of *AgrPMT*, *AgrPME1* and *2*, and *AgrPMEI* genes in clusters. (**C**) Visualization of coexpression networks, with the top 10 genes shown (IDEP 2.01, soft threshold 5). Red circles designate *AgrPMTs* that are coexpressed with genes encoding other RG-I biosynthetic enzymes.

In the celery genome, we identified all genes with the PF03141 domain (pectin methyltransferases, PMTs) and analyzed their expression. Alongside PMTs, the expression of genes encoding PMEs (types 1 and 2) and PMEIs was also analyzed. Cluster analysis revealed that pectin biosynthesis, including methylesterification and subsequent demethylesterification, primarily occurs during the early developmental stages. The celery genes encoding pectin-modifying enzymes (PMT, PME) and PMEI were predominantly divided into two networks: those upregulated in vascular bundles (cluster 1) and those upregulated in young collenchyma (clusters 5 and 7) (Figure 10). Most *AgrPMT* genes were upregulated in young tissues, peaking in collenchyma (clusters 5 and 7). Notably, some *AgrPMTs* were coexpressed with genes encoding RG-I biosynthetic enzymes (AgrRRT and AgrGALS2) (Figure 10C), suggesting that these PMTs may play a role in the methylesterification of RG-I.

#### 2.5.4. *O*-Acetylation of Cell Wall Polysaccharides

In addition to methylesterification, *O*-acetylation is a common modification that plant cell wall polysaccharides, such as hemicelluloses and pectins, undergo [58,59]. Two protein families, RWAs and TBLs, localized in the Golgi apparatus, act as acetyltransferases [18]. RWAs acetylate xylans [60], while TBLs have been described to acetylate various substrates, including xylan, xyloglucan, mannan, and pectins [18]. Acetylester bonds at *O*-2 and/or *O*-3 of GalA residues are hydrolyzed by pectin acetylesterases (PAE) [9,61]). There is experimental evidence that some members of the GDSL esterase/lipase protein family also function as deacetylases [20]. This family belongs to a subclass of lipolytic enzymes with a broad substrate specificity, capable of catalyzing acyl group transfer in hydrolase reactions involving both lipid and non-lipid substrates [62]; for certain members, a key role in cutin deposition has also been demonstrated [63].

**Figure 11:**
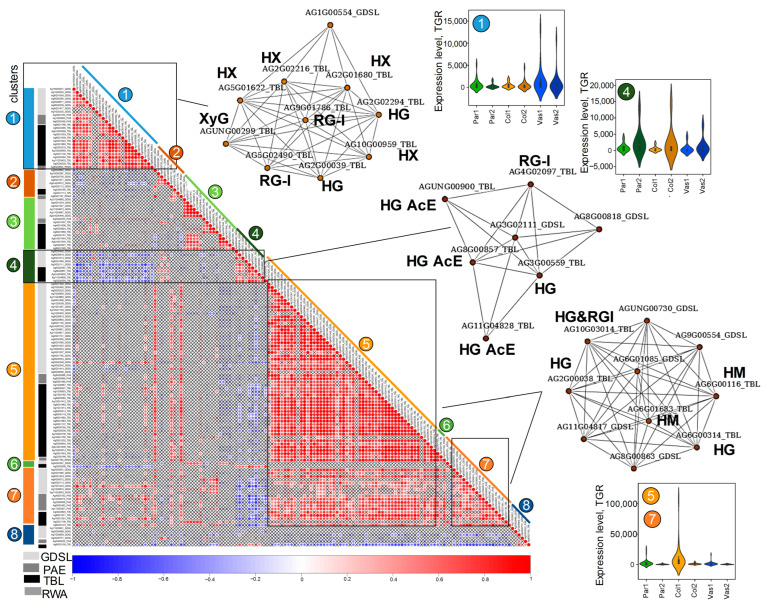
Correlation between the expression of genes encoding acetyltransferases (TBLs, RWAs) and esterases (PAEs) in celery tissues with the clustering of genes related to cell wall proteins (in circles). Genes that are positively coexpressed with others are marked by red dots, while those that are negatively coexpressed are marked by blue dots. Black frames designate large coexpressed gene groups. The size and color of the dots reflect the absolute value of Pearson’s correlation coefficient (*p* < 0.01; insignificant *p*-values are marked with X, Table S5). Cluster numbers are given in circles using the same color scheme as in Figure 7. These clusters include genes that are upregulated in designated tissues: cluster 1—Vas1; 2—Col1/2; 3—Par1; 4—Par2; 5—Col1; 6—Par1/2; 7—young/Col1; 8—mature/Vas2. Par—parenchyma; Col—collenchyma; Vas—vascular bundles. Coexpression networks for clusters 1, 4, and 5/7 were visualized, showing the top 10 genes (IDEP 2.01, soft threshold 5). The dynamics of gene expression in these clusters are illustrated in violin plots. Red circles indicate *PMTs* that are coexpressed with genes encoding other RG-I biosynthetic enzymes. HX—heteroxylan; HM—heteromannan; HG—homogalacturonan; RGI—rhamnogalacturonan I; AcE—acetylesterase activity.

Similar to the genes for enzymes involved in (de)methylesterification, the genes for enzymes associated with (de)acetylation were categorized into two main networks: those upregulated in young vascular bundles (cluster 1) and those upregulated in young collenchyma (clusters 5 and 7; Figure S3). Notably, several genes in cluster 4 exhibited a negative correlation with genes from clusters 1 and 7, as they were upregulated in mature tissues (Figure 11). *AgrTBLs* within this network are homologs of the gene for atypical acetyltransferase AtTBL38, which localizes to the cell wall microdomains of mucilage secretory cells and functions as an acetylesterase [64]. We hypothesize that the identified homologs of TBL38 in celery may exhibit similar enzymatic activity.

Genes encoding TBLs that potentially acetylate xyloglucan were activated in young parenchyma (cluster 3, Figure S3), while genes for pectin-specific TBLs were notably activated in young collenchyma (Figure S3). In cluster 1, among the top 10 coexpressed genes, 5 were genes for TBLs that acetylate xylan, alongside genes for TBLs that acetylate RG-I and HG. Additionally, this network included a gene for a GDSL esterase (Ag1G00554), likely involved in xylan deacetylation; this gene is an ortholog of *AT1G09390*, which is itself an ortholog of a rice gene encoding a putative Deacetylase on ARabinosyl sidechain of Xylan 1 (DARX1) [65]. In the coexpression network combining clusters 5 and 7, the top 10 coexpressed genes included those encoding TBLs that potentially acetylate mannans and pectins (Figure 11). All networks encompass genes for enzymes involved in both acetylation (TBLs, RWAs) and deacetylation (PAEs, GDSLs). The GDSL esterase/lipase family is one of the largest groups, comprising 66 genes, nearly half of which were upregulated in young collenchyma (clusters 5 and 7). The top 10 coexpressed genes in the “collenchyma” network included five *AgrGDSLs*. While there is no direct evidence that all members of the GDSL esterase/lipase family function as deacetylases, it is plausible that some are involved in the deacetylation of non-cellulosic polysaccharides, warranting further investigation into this protein group.

#### 2.5.5. Mannan Biosynthesis and Modifications

**Figure 12:**
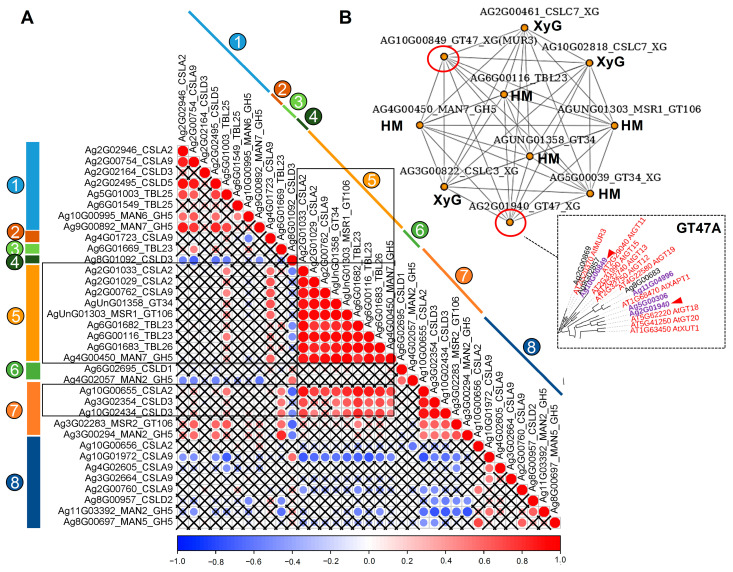
(**A**) Coexpression of genes encoding mannan-related enzymes according to their clustering (in circles). The black frames highlight coexpressed genes from clusters 5 and 7. Genes exhibiting positive coexpression with others are marked by red dots, while those that show negative coexpression are marked by blue dots. The size and color of the dots reflect the absolute value of Pearson’s correlation coefficient (*p* < 0.01; insignificant *p*-values are indicated with X, Table S5). Cluster numbers are given in circles using the same color scheme as in Figure 7. These clusters include genes that are upregulated in designated tissues: cluster 1—Vas1; 2—Col1/2; 3—Par1; 4—Par2; 5—Col1; 6—Par1/2; 7—young/Col1; 8—mature/Vas2. Par—parenchyma; Col—collenchyma; Vas—vascular bundles. (**B**) Visualization of gene coexpression network for xyloglucan-related (XyG), heteromannan-related (HM), and GT47 proteins, showing the top 10 genes (IDEP 2.01, soft threshold 5), along with a portion of the phylogenetic tree of the GT47 family (clade A); the full version is presented in Figure S4. Red arrows on the fragment of the phylogenetic tree fragment and red circles on the coexpression network indicate potential galactosyltransferases involved in HM and XyG biosynthesis.

Mannans are relatively minor components of dicot plant primary cell walls [66]; however, a significant upregulation of genes encoding enzymes involved in mannan biosynthesis and modifications was observed in collenchyma (Figure 12). Within the coexpression network, four isoforms of Cellulose Synthase-Like A (AgrCSLA) were identified, along with two isoforms of AgrCSLD3 and AgrMSR (GT106) (Figure 12A). It has been proposed that MSRs interact with CSLAs to modulate (gluco)mannan elongation [67]. Additionally, the gene for MAGT1 (GT34), which encodes MUCI10, a mannan α-1,6-galactosyltransferase [68], was co-expressed with the aforementioned synthases. Although genes encoding enzymes orthologous to MBGT (mannan β-galactosyltransferase, GT47) were not expressed in celery tissues, four genes encoding members of GT47 (clade A) were upregulated in collenchyma; all of these genes were orthologs of Arabidopsis genes encoding xyloglucan galactosyltransferases (Figure S4). In many eudicots, β-galactoglucomannans (β-GGM) are believed to function similarly to xyloglucan in primary cell wall-rich tissues [69]. Based on this assumption, we suggest that other members of the GT47 (clade A) family, originally known for their activity as xyloglucan galactosyltransferases, may also accommodate GGM as an acceptor.

Through phylogenetic analysis of celery and Arabidopsis GT47 sequences, along with the described activities of several glycosyltransferases in family 47 [70,71], we propose the following activities: Ag5G00306 and Ag2G01940 (AgrXLT2, AT5G62220), Ag10G00849 (AgrMUR3, AT2G20370), and Ag11G04996 (AT4G22580) may function as xyloglucan or mannan galactosyltransferases. Probable substrate specificity for other celery GT47 is presented in Figure S4.

Three potential *O*-acetyltransferases, which are orthologs of TBL23 and TBL26 [72], along with endo-β-mannanase 7 (GH5), were also part of the coexpression network. Based on the coexpressed set of mannan synthase and acetylase genes, we hypothesize that mannose residues in the mannan backbone are synthesized with *O*-acetylated. This backbone comprises glucose residues and is decorated with galactose side chains, forming acetylated galactoglucomannan. Despite the identification of relatively few genes encoding mannan-related proteins, their high activity at the transcriptome level indicates a significant role for heteromannans in the collenchyma cell wall structure, even though they represent a minor component. The enzymes responsible for the deacetylation of mannose residues remain unidentified, but their discovery could have substantial implications for industrial applications. A high degree of mannan acetylation contributes to its recalcitrance [73], which could be exploited to modify polysaccharide properties for various industrial purposes [74]. Our data demonstrate that mannan *O*-acetyltransferase genes (*AgrTBL23*, *AgrTBL26*) co-express with several *AgrGDSL* genes. Although there is currently no experimental data to establish substrate specificity for these GDSL proteins, they are promising candidates for mannan deacetylases, requiring further study.

#### 2.5.6. Xylan Biosynthesis

**Figure 13:**
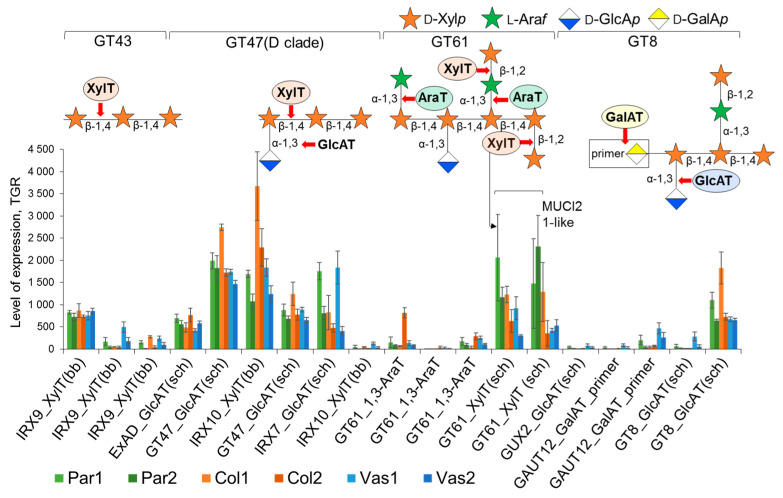
Expression of genes for glycosyltransferases involved in xylan biosynthesis (GT43, 47, 61, 8) in celery tissues at two developmental stages (young (1) and mature (2)). bb—backbone; sch—side chain; T—transferase; Par—parenchyma; Col—collenchyma; Vas—vascular bundles. A list of genes is provided in Table S4.

Along with heteromannans, small amounts of heteroxylans were also found in celery cell walls [2,8]. Based on the set of genes encoding glycosyltransferases involved in xylan biosynthesis, we propose that xylan decorated with glucuronic acid residues is actively synthesized in celery primary cell walls. The substitution with arabinose residues appears to be less pronounced; however, one gene encoding arabinosyltransferase was specifically activated in mature collenchyma (Figure 13). Furthermore, glucuronoxylans in celery are likely to exhibit high branching due to xylan side chains. Notably, celery genes homologous to *AT2G41640* (GT61) were especially upregulated in the parenchyma and young collenchyma tissues (Figure 13). AT2G41640 is closely related to MUCI21, known as a seed mucilage xylosyltransferase that facilitates the addition of β-1,2-xylose units to the xylan backbone [75]. The fact that heteroxylans in collenchyma cell walls are highly substituted is indirectly confirmed by the absence of labeling with the LM10 antibody [3], which specifically recognizes unsubstituted β-1,4-xylan [34].

## 3. Discussion

### 3.1. Celery Parenchyma and Collenchyma Cell Walls Are Enriched with Pectins

Celery cell walls are known to be rich in pectins [8,76]. Our data indicate that pectins comprise approximately 50% of the cell walls in young collenchyma and 42% in young parenchyma. Previous studies reported a similar pectin content (51%) in celery parenchyma, primarily considering only RG-I with galactan and arabinan side chains as pectins while omitting HG from the calculations of total pectin content [8,76]. In contrast, our findings show that RG-I with galactan and arabinan side chains constitutes 13% of the cell walls in parenchyma and 17% in collenchyma, with HG content around 30% in both cell types. Additionally, our biochemical and transcriptomic data do not support the notion that collenchyma walls have lower proportions of α-(1,5)-l-arabinan and β-(1,4)-d-galactan side chains of RG-I [2,6,8]. It has been reported that celery collenchyma cell walls contain lower proportions of pectic polysaccharides and higher proportions of cellulose, xyloglucan, and heteroxylan compared to celery parenchyma cell walls [2]. However, according to our data, the pectin content in collenchyma cell walls is comparable to that in parenchyma cell walls, or even slightly higher, with a more pronounced decrease in pectin content observed in parenchyma at the mature stage. Moreover, a set of genes encoding homogalacturonan biosynthetic enzymes was activated in the collenchyma at the young stage compared to other tissues (Figure 8). At the mature stage, RG-I content slightly decreases in both tissues, while HG content decreases more significantly in parenchyma cell walls. The reduction in HG labeling in cross-sections of collenchyma, despite stable HG levels as measured biochemically, may be attributed to the masking of HG epitopes. This masking could result from the reorientation and tighter packing of cellulose microfibrils, along with potential interactions with RG-I and other hemicelluloses such as xyloglucan and mannans. A similar epitope masking may occur with the branched galactan side chains of RG-I. However, the partial degradation of RG-I side chains cannot be ruled out, as there is a noted decrease in arabinose and galactose content in the pectin fraction of mature cell walls (Figure 2A). Notably, the accumulation of transcripts for polysaccharide-degrading enzymes (such as pectate lyases and hydrolases) was primarily observed during the early developmental stage. The significant reduction in pectin content in mature parenchyma (Figure 2B) may be associated with increased cell wall rigidity. Previous studies have shown that the removal of the pectic matrix from celery parenchyma cell walls leads to microfibril swelling and aggregation [76]. Our study also confirmed that collenchyma walls contain higher proportions of xyloglucans [3].

A significant portion of the upregulated genes in collenchyma encodes enzymes involved in RG-I biosynthesis and modifications. Through coexpression network analysis, we identified genes encoding enzymes that may be involved in the biosynthesis of RG-I and hypothesized the existence of a complex necessary for synthesizing the backbone of this polysaccharide. Furthermore, we propose that these complexes may be tissue-specific: Parenchyma and collenchyma RG-I with galactan side chains might be synthesized by different sets of isoforms. Similar complexes have been characterized for other cell wall polysaccharides, such as xylan [77] and HG [53]. The upregulation of genes encoding several RRT isoforms has been observed in flax fibers during the formation of the thickened (tertiary, or G-type) cell wall [78,79]. However, the potential involvement of various RGGAT isoforms in RG-I biosynthesis in collenchyma, as well as the possible role of PMTs in methylesterification of RG-I, has not been previously reported.

Interestingly, within the coexpression network, only a single gene isoform for galactan synthase (GALS2) was found alongside multiple members encoding RRTs, OFUTs, and GT116s. It has been shown that Arabidopsis AtGALS1 functions as a bifunctional enzyme, catalyzing both the transfer of galactose from UDP-α-d-Gal and the transfer of arabinopyranose from UDP-β-l-Ara*p* to galactan chains [80]. This discovery suggests that plants can achieve structural diversity in polysaccharides without requiring a specific glycosyltransferase for each type of glycosidic linkage. Based on this assumption, it is plausible that AgrGALS2 may be involved in the synthesis of branched galactan chains substituted with β-(1→6)-Gal*p*, although it was shown that branched galactan substituted with an α-(1→6)-Ara*f* at the *O*-4 Gal position (Gal_6_Ara_1_) is not a substrate for AtGALS1 [80]. Furthermore, we speculate that the numerous *AgrOFUTs* activated in collenchyma may encode proteins that are involved in the biosynthesis of branched RG-I. The (1→6)-branched β-(1→4)-d-galactans, which we identified as predominant in collenchyma, have previously been revealed in hemp and flax sclerenchyma fibers [81,82], which are characterized by thickened cell walls and are also specialized for mechanical functions.

Immunolabeling analysis did not reveal significant differences in the levels of low- and high-esterified HG between parenchyma and collenchyma cells at different developmental stages. In both tissues, HG content decreased at the mature stage; however, in parenchyma cells, low-esterified HG was primarily concentrated in the cell junctions (Figure 3). Specific sets of upregulated genes for PMEs, PMEIs, and PAEs were identified in all tissue types, including vascular bundles, parenchyma, and collenchyma, with many of these genes being upregulated primarily during the early developmental stage. Previous studies indicated that the degree of methylesterification of HG in collenchyma cell walls is lower than that in parenchyma [2]. This lower methylesterification is thought to be associated with the rheological properties of pectins, facilitating the formation of semi-rigid gels through interactions between Ca^2+^ ions and pectic carboxyl groups. Furthermore, the character of demethylesterification (whether it occurs in a block-wise or random manner) can have contrasting effects on the mechanical properties of the cell wall, either enhancing rigidity or promoting loosening [13,14]. During the early stage of active elongation in collenchyma cells, it is hypothesized that demethylesterification occurs in a random manner. Although the specific extent of RG-I methylesterification has not been established, we have identified several *PMT* genes within the coexpression network of genes encoding RG-I biosynthetic enzyme. Typically, GalA residues in the RG-I backbone are not methylesterified [83], although they may be highly acylated at *O*-2 and/or *O*-3. However, studies have shown that in flax, 17–40% of GalA residues in RG-I are methylesterified [84], and 22% of GalA residues are methylesterified in tobacco RG-I [85]. The possibility of methylesterification of other residues besides GalA, as well as the potential for methylesterifing other substrates (such as hemicelluloses) alongside pectins, cannot be ruled out. Information on PMT substrate specificity and mechanisms of action is extremely limited, which is surprising given the significant impact that the removal of methyl groups has on cell wall properties. The enrichment in pectins, especially branched RG-I, likely increases the “water sensitivity” of collenchyma cell walls, which contributes to their flexibility and supports wall elongation. Additionally, the acetylation of pectins significantly affects their rheological properties [18].

### 3.2. Celery Parenchyma Cell Walls Are Enriched with Cellulose, but Collenchyma Cellulose Is More Ordered

Celery parenchyma cell walls contain more cellulose compared to collenchyma (as shown in this study and in previous research [36]), with cellulose content increasing as the cells mature (Figure 2B). Despite this, collenchyma cell walls exhibit more intense labeling with CBM3a, which targets crystalline cellulose [35], compared to parenchyma cell walls (Figure 4). Previous studies reported that celery collenchyma contains high amounts of amorphous cellulose [86]. However, the cellulose in collenchyma is more homogeneously oriented, with slightly larger crystallites (2.4–3.6 nm, [87]) compared to those found in typical primary cell walls, including celery parenchyma (2–3 nm, [8]). In a study examining the binding capacity of different cellulose-binding CBMs, it was found that celery collenchyma preferentially bound type B CBMs (CBM4-1, 17), which target internal regions of amorphous cellulose, unlike the binding observed in celery parenchyma walls [35]. Binding was most effective in the inner regions of the thickened collenchyma cell walls, and removing pectin (via pectate lyase treatment) enhanced CBM17 binding to collenchyma walls and extended it to parenchyma walls. The authors concluded that celery collenchyma cell walls do not form highly ordered crystalline structures. However, they also demonstrated that CBM3a (type A), which targets crystalline cellulose, binds to all celery primary cell walls, with stronger fluorescence observed in collenchyma compared to parenchyma walls.

### 3.3. Xyloglucan Is a Crucial but Not Dominant Polymer in Collenchyma Cell Walls

It is a well-established concept that primary cell walls (type I) in eudicots and non-commelinoid monocots are built on the cellulose–xyloglucan network embedded in a pectin matrix [7]. In celery, parenchyma is characterized by a low xyloglucan content [8], leading to the proposal that this amount may not be sufficient to coat the cellulose microfibrils [8]. The xyloglucan content in celery collenchyma is about twice that in the parenchyma, but still lower than in the “typical” primary cell walls of dicotyledonous angiosperms like Sycamore or *A. thaliana*, where xyloglucan comprises about 20–25% [88], making it more comparable to that found in grasses (2–5%).

Despite this, genes encoding all characterized xyloglucan biosynthetic enzymes are expressed in all analyzed celery tissues, with significant upregulation observed in young collenchyma. This suggests a notable intensification of xyloglucan biosynthesis in collenchyma tissue. At the mature stage, the expression of these biosynthetic genes decreases across all tissues. Xyloglucan modifications occur in both young and mature collenchyma. Two *XTHs* rank among the top 10 genes with the highest expression levels in collenchyma, with more than 370,000 (*AgrXTH6*) and 320,000 (*AgrXTH7*) TGR in Col1 (Tables S2 and S4); *AgrXTH6* maintains high expression in Col2 (Table S2). Both genes belong to group I of XETs, for which homo- and heterotransglycosylase activity has been demonstrated for products of orthologous genes [21]. Interestingly, the expression of genes encoding enzymes involved in xyloglucan acetylation (TBLs; Figure S3) suggests that xyloglucan in parenchyma cell walls can be synthesized in a more heavily acetylated form compared to xyloglucan in collenchyma.

Furthermore, it is hypothesized that there may be an interaction or association between xyloglucans and pectins in the collenchyma cell wall. This is indirectly indicated by the observation that partial pectin removal (as shown in cross-sections, Figure 5) leads to a decrease in the intensity of xyloglucan labeling. In parenchyma, this effect is less pronounced. We propose that interactions between xyloglucan and pectins (and possibly other hemicelluloses) are regulated by XTH and that the rearrangement of these interactions plays a crucial role in cell wall changes during growth and functioning. It has been shown that more than 30% of xyloglucans in suspension cultures is covalently bound to pectins [89,90]. Given that celery cell walls are enriched with pectins, we hypothesize that pectins, rather than xyloglucan, coat the cellulose microfibrils in celery cell walls, regulating the spacing between microfibrils, their interactions, and possibly their crystallinity. This assumption is further supported by the enhanced binding of CBM type B to cellulose after pectin removal (as noted above). Nevertheless, even a small amount of xyloglucan can significantly impact the arrangement of the pectin–cellulose network.

### 3.4. O-Acetylated Galactoglucomannans in Collenchyma Cell Walls May Have Overlapping Functions with Xyloglucan

A notable feature of collenchyma cell walls is the presence of mannans. Immunolabeling studies have shown that mannans are more intensely labeled in parenchyma cell walls than in collenchyma, likely due to the more accessible epitopes in parenchyma, which allow for stronger antibody binding. In our previous work, we observed an increased mannan content in young collenchyma based on monosaccharide yield, although this was not detected using immunodot analysis. Furthermore, we found that genes involved in mannan biosynthesis and its modifications are upregulated in collenchyma compared to parenchyma [21].

In the current study, we identified a more comprehensive set of mannan synthase genes *(CSLA2*, *9*, *CSLD3*, *GT34*, and *GT106*) and confirmed the upregulation of genes encoding enzymes involved in mannan biosynthesis and modifications at the early stage of collenchyma development, followed by a decrease at later stages. Based on the coexpression of mannan-related glycosyltransferases and modifying enzymes, we concluded that *O*-acetylated β-galactoglucomannans (Acβ-GGM), with a backbone composed of [4)-Man-β-(1,4)-Glc-β-(1] units, are present in collenchyma cell walls, with Man residues primarily substituted by α-Gal. Interestingly, in Arabidopsis tissues, there are few or no α-Gal side chains on AcGGM [91]. Several GT47A-encoding genes were found to be coexpressed with genes encoding xyloglucan and mannan biosynthetic enzymes (Figure 12B and Figure S4). However, further investigations are required to determine their specific substrate activity.

## 4. Materials and Methods

### 4.1. Plant Materials

Celery plants (*Apium graveolens* L., var. *secalinum* Yablochny) were grown in pots with a soil layer of 50 cm in a greenhouse maintained at 22–24 °C under artificial lighting with a 16 h day length and daily watering. For light microscopy and immunocytochemistry, petiole segments, 1 cm in length above the leaf sheath, were used. For transcriptomic analysis, tissues enriched with collenchyma (Col), parenchyma (Par), and vascular bundles (Vas) were manually separated from a 4 cm section of the celery petiole, receding 1 cm from the leaf sheath. Additionally, sections containing excised collenchyma strands were collected, along with collenchyma-free samples (CF, Figure 1). Samples were collected at the same level (1 cm from leaf sheath) during two growth stages: young (30–40 days after cotyledon emergence, designated as “1”) and mature (80–90 days after cotyledon emergence, designated as “2”). The samples were immediately frozen in liquid nitrogen and stored at −80 °C until analysis. RNA-Seq analysis was conducted on all samples from each growth stage in four biological replicates.

### 4.2. Light Microscopy

Morphological parameters of collenchyma cells, including cell count on cross-sections, the area occupied by cell walls, and the mean length of individual macerated cells, were measured from images obtained using a light microscope (Axioscope A1, Zeiss, Gottingen, Germany). Maceration was conducted using 0.01% pectinase from *Aspergillus niger* (Sigma-Aldrich, Saint Louis, MO, USA, CAS no. 9032-75-1) at 30 °C for 30 min. The analysis was performed using Image Tool 3.0 and ImageJ 1.52a software. The proportion occupied by collenchyma in the cross-section was estimated by calculating the sum of the areas of all collenchyma strands relative to the total petiole area. This estimation was performed in pixels using ImageJ software. For the analysis, sections from the same area of the petiole used for biochemical and transcriptomic analyses were selected. A total of six petioles from different plants were used for each developmental stage.

### 4.3. Cell Wall Fractionation, Polysaccharide Yield, and Monosaccharide Analysis

Before analysis, collenchyma and parenchyma tissues were washed with 95% ethanol to remove pigments. The samples were ground in liquid nitrogen and homogenized in a cold 0.01 M NaOAc buffer (pH 5.0–5.2, 4 °C), followed by several washes with water. The resulting pellet was washed three times with 80% (*v*/*v*) ethanol, once with 80% (*v*/*v*) acetone, and then dried. Cell wall polymers were sequentially extracted with 1% (*w*/*v*) ammonium oxalate (pH 5) in a boiling water bath for 1 h, repeated twice. After washing with water and drying, the samples were treated with 4 M KOH containing 3% H_3_BO_4_ for 12 h, followed by an additional 24 h. The KOH fractions were neutralized with acetic acid to pH 7 (according to [22] with modifications). Fractions extracted with ammonium oxalate (AO) and alkali (KOH) were desalted by passing through a Sephadex G-25 column (15 × 60 mm) and then dried. Non-cellulosic polysaccharides in cellulose-enriched residues, treated with chelator and alkali, were quantified as the sum of pectins and hemicelluloses released after hydrolysis of the residues with trifluoroacetic acid (TFA).

For monosaccharide analysis, dried samples were hydrolyzed with 2 M TFA at 120 °C for 1 h, dried again to remove TFA, and dissolved in deionized water. The monosaccharide composition was analyzed using high-performance anion-exchange chromatography with a DX-500 system (Dionex, Sunnyvale, CA, USA), employing a CarboPac PA-1 column (4 × 250 mm, Thermo, Waltham, MA, USA) and a pulsed amperometric detector ED40 (Waveform A, 500 ms). Neutral monosaccharides were separated isocratically with 15 mM NaOH, while uronic acids were separated using a linear gradient from 23.6 mM NaOH in 0.1 M NaOAc to 40.6 mM NaOH in 0.3 M NaOAc over 10 min. The column temperature was maintained at 30 °C, with a flow rate of 1 mL min^−1^. Monosaccharide standards for calibration were treated similarly with 2 M TFA. Results were analyzed using PeakNet 4.30 software [92].

The polysaccharide yield in each fraction was calculated by summing the monosaccharide content [93]. The hemicellulose yield was determined by summing the monosaccharides that comprise the XXFG fragment of xyloglucan (glucose, xylose, galactose, and fucose in a 4:3:1:1 ratio [94]) along with mannan. The pectin yield was estimated based on the total content of rhamnose and galacturonic acid. Additionally, the content of arabinose content and the portion of galactose not attributed to the XXFG fragment of xyloglucan were included in this estimation. The content of HG was determined by subtracting the amount of galacturonic acid associated with RG-I from the total galacturonic acid content. The proportion of galacturonic acid in the RG-I backbone was estimated to be equivalent to the rhamnose content. In calculating the proportions of arabinose and galactose associated with RG-I and arabinogalactans, we referenced previously published data on the ratios of these components in the cell walls of collenchyma and parenchyma tissues [6,8]. Before this, the proportion of galactose attributed to xyloglucan was deducted from the total galactose content. The amount of mannan was determined by measuring the mannose content across all fractions and cellulose-enriched residues. The xylan content was estimated as the difference between the total xylose content in the cell wall and the xylose content attributed to the XXFG fragment of xyloglucan. Finally, the cellulose yield was calculated as the amount remaining after the removal of pectins and hemicelluloses. The analysis was performed for four independent biological replicates. Results from this analysis are presented in the diagrams as the mean ± SD (standard deviation).

### 4.4. Immunocytochemistry

The celery parts (5 mm) were cut from 1 cm above the leaf sheath using a blade, then embedded in agar blocks and sectioned at 100 µm with a vibratome VT1000S (Leica Biosystems, Nussloch, Germany). For immunohistochemical detection, the sections were incubated in Tris-buffered saline (TBS, pH 7.5) with 2% (*w*/*v*) bovine serum albumin (BSA) for 1 h to block non-specific binding. The sections were subsequently incubated for 1.5 h with primary antibodies LM5, LM6, LM11, LM21, LM25, LM26, and PAM1 at a dilution of 1:5; INRA-RU2 at a dilution of 1:3; and CBM3a at a dilution of 1:100 (Table S1). Secondary antibodies, anti-rat IgG, and anti-mouse IgG conjugated to fluorescein-5-isothiocyanate (FITC, Sigma-Aldrich, St. Louis, MO, USA, CAS no 3326-32-7), were used at a dilution of 1:100, and the samples were incubated for 1 h in the dark. All antibodies were diluted in TBS. Control experiments were performed by omitting the primary antibody (Figure S5). To remove the masking of cross-linking glycan epitopes by pectins, 0.1% pectinase from *Aspergillus niger* (Sigma-Aldrich, Saint Louis, MO, USA, CAS no. 9032-75-1) was applied prior to immunolabeling. Sections were examined using a laser confocal fluorescence microscope (LSM 510 Meta, Carl Zeiss, Jena, Germany). Immunofluorescence was detected with excitation at 488 nm and emissions at 503–550 nm. The transmitted light channel was used to observe anatomical details. Fluorescence intensity was quantified using Image Tool 3.0 and ImageJ 1.52a software. When evaluating the intensity of labeling, only the green channel intensity was measured after RGB color separation, with a minimum value of 0 and a maximum value of 254. To ensure valid comparisons of labeling between collenchyma and parenchyma cells with varying cell wall thicknesses, a minimum of 30 fragments (for thin-walled parenchyma cells) and 50 fragments (for thick-walled collenchyma cells) of 4 × 4-pixel cell wall regions were analyzed per image. The size of the selected fragment was chosen to cover the entire thickness of the parenchyma cell wall in areas where it does not contact neighboring cells, specifically targeting the region of the cell wall stack at the intercellular spaces. The number of analyzed cross-sections ranged from 4 to 9 (Figure S6). Statistical significance was determined using the Student’s *t*-test, with a *p*-value of less than 0.05 considered significant. Characteristics of the antibodies, including epitope specificity, are detailed in Table S1.

### 4.5. RNA Isolation, Sequencing, the Analysis of Differentially Expressed Genes

RNA extraction was performed using the “RNeasy Plus Universal Mini Kit” (Qiagen, Hilden, Germany). The concentration and integrity of the RNA were assessed through agarose gel electrophoresis and with a NanodropND-1000 spectrophotometer (Thermo Fisher Scientific, Wilmington, DE, USA). Further evaluation of RNA integrity was conducted using an Agilent 2100 Bioanalyzer (Agilent Technologies, Waldbronn, Germany). RNA sequencing was performed on an Illumina HiSeq 6000 (Illumina, San Diego, CA, USA) at Novogene in Europe (https://en.novogene.com/, accessed on 20 August 2024) using 150 bp paired-end reads. The analysis of RNA-Seq data, provided as pools of raw reads in fastq files for each sample, was conducted in several stages. Initial quality assessment and filtering for sequences of rRNAs, tRNAs, snRNAs, and snoRNAs (https://rnacentral.org/, accessed on 20 August 2024) were performed using the FastQC program and BBDuk algorithms from the BBTools v38.73 package (BBTools, https://sourceforge.net/projects/bbmap/, accessed on 20 August 2024). Clean reads were then mapped to the celery genome, and gene expression was quantified using the HISAT2 and StringTie algorithms [95] with parameters for paired reads. The celery genome (*A. graveolens*) and the corresponding annotation file were downloaded from the BIO2DB celery genome database (CGD: http://celerydb.bio2db.com, accessed on 20 August 2024). Expression levels were represented as total gene reads (TGRs), calculated as the sum of all reads mapped to the coding region of each gene. Normalization of TGR values was achieved by calculating a geometric mean for each gene across all samples, and differential expression analysis with false discovery rate adjustment (padj) was performed in R using the DESeq2 package ([96], https://www.r-project.org, accessed on 20 August 2024). A gene was considered expressed if the normalized TGR values in at least one sample were ≥16 [97]. Following the initial PCA analysis, several replicates that exhibited significant differences compared to the remaining three were filtered out. Ultimately, for further normalization, we used a total of 29 samples: Par1 (4 replicates), Par2 (4 replicates), Col1 (3 replicates), Col2 (3 replicates), CF1 (4 replicates), CF2 (3 replicates), Vas1 (4 replicates), and Vas2 (4 replicates). Differential expression was determined based on three criteria: TGR ≥ 16 in at least one sample, a twofold or greater change in TGR values when comparing samples pairwise, and padj ≤ 0.01. The gene expression values (TGR), results of the differential expression analysis, and functional annotations of celery genes according to results of blast+ 2.2.28 ([98], https://bioweb.pasteur.fr/packages/pack@blast+@2.2.28, accessed on 20 August 2024) against Arabidopsis and online protein annotation tool Mercator4 ([99], https://www.plabipd.de/mercator_main.html, accessed on 20 August 2024) can be found in Table S2.

### 4.6. Bioinformatics Analysis

Clustering was performed using Morpheus software (https://software.broadinstitute.org/morpheus, accessed on 20 August 2024) employing the K-Means Clustering Algorithm. Gene ontology (GO) analysis was carried out for Arabidopsis genes orthologous to upregulated celery genes using ShinyGO v0.741 ([100], http://bioinformatics.sdstate.edu/go/, accessed on 20 August 2024) with a false discovery rate (FDR) cutoff of <0.05. The list of genes, their expression, and distribution across clusters are provided in Table S2 (common clustering) and Table S4 (genes encoding cell wall-related proteins). Protein annotation was conducted using the CAZy ([9], http://www.cazy.org/), UniProt ([37], https://www.uniprot.org/), Phytozome ([38], https://phytozome-next.jgi.doe.gov/), and TAIR ([39], https://www.arabidopsis.org/) databases (all accessed on 20 August 2024), supplemented with data from the literature. The top 10 GO categories and the upregulated genes within common clusters are summarized in Table S3.

For phylogenetic analysis, the amino acid sequences of celery and Arabidopsis gene families were aligned using the M-Coffee web-based service ([101], https://tcoffee.crg.eu/, accessed on 20 August 2024). The sequences used in the phylogenetic trees, along with their gene symbols and brief descriptions, are listed in Table S6. Truncated sequences were excluded from the analysis. The alignment was subjected to a maximum-likelihood phylogenetic analysis using IQTREE software ([102], version 2.2.2.6). The best-fit models of sequence evolution were selected based on the Bayesian Information Criterion, as implemented in ModelFinder [103]. Ultrafast bootstrap branch support [104] with 10,000 replicates was used to construct the dendrograms, with values below 95 considered non-significant. The unrooted tree was visualized using iTOL 6.5.2 [105]. For constructing correlation coefficient plots and violin plots, the SRplot online platform was utilized [106], with a significance level set at 0.01. The correlation coefficient values for the visualized lower triangle networks are provided in Table S5. Coexpression analysis was performed using data from samples Par1,2, Col1,2, Vas1,2, and CF1,2. Coexpression subnetworks were constructed and visualized using iDEP 2.01 software ([107], http://bioinformatics.sdstate.edu/idep/, accessed on 20 August 2024).

RNA-Seq data were validated using qPCR for four selected genes. The procedure was described in our previous study [21]. The gene for elongation factor Tu (Ag9G01513) served as the reference gene. The list of genes, primer sequences, and results of verification are provided in Figure S1.

## 5. Conclusions

Celery parenchyma and collenchyma cell walls have long intrigued plant scientists due to their unique and unconventional characteristics. Despite several studies on the biochemistry of celery collenchyma and parenchyma cell walls, significant knowledge gaps remain regarding various processes. These include the relationship between polysaccharide structures and their functions, the impact of modifying groups on these functions, and the regulation of these processes at the molecular–genetic and biochemical levels.

A particular strength of the current study lies in its transcriptome analysis, which was performed on samples enriched in specific tissue types: parenchyma, collenchyma, and vascular bundles. This approach allowed us to link transcriptome data with biochemical analyses and to identify specific isoforms among members of multigene families, whose products may be involved in the formation and modification of polysaccharide substrates.

In each celery tissue type, we observed a pool of upregulated genes for enzymes involved in the methylesterification and *O*-acetylation of pectins, as well as their removal. Notably, young collenchyma is significantly enriched with transcripts of methyl and *O*-acetyltransferases, along with transcripts of RG-I biosynthetic enzymes, which likely act as a complex. This corresponds to an increased content of RG-I in collenchyma cell walls. Additionally, we found that (1→6)-branched β-(1→4)-d-galactan chains are more pronounced in collenchyma cell walls than in parenchyma cell walls. Given the established sets of expressed glycosyltransferases, we infer that *O*-acetylated galactoglucomannans and *O*-acetylated branched glucuronoxylans are present in celery cell walls. By utilizing transcriptomic data from celery petiole tissues, we can propose potential substrates for PMT, TBL, and GDSL proteins based on their gene coexpression networks. The data from this study provide a limited number of specific candidate genes for further investigation into collenchyma cell wall development through molecular–genetic approaches, such as the generation of transgenic plants with altered gene expression or genome editing techniques to modulate cell wall biosynthesis and modification.

## Figures and Tables

**Table 1:** A list of protein families involved in the biosynthesis and modification of primary cell wall polysaccharides, along with their probable activities and the number of genes (nG) expressed in celery petiole tissues. T—transferase; E—esterase (methylE, acetylE). XyG—xyloglucan. RGI—rhamnogalacturonan I. HG—homogalacturonan. AGP—arabinogalactan protein. Full names for abbreviations of protein family names (GH, GT, CESA, etc.) are provided in the main text or can be found in CAZY [9].

Potential Substrate	Biosynthesis		nG	Modifications		nG
Cellulose	GT2 (CESA)	PF13632, PF14569	13	GH9; Endoglucanase	PF00759	20
XyG	CSLC3/7	PF13632	6	GH16; XTH	PF06955, PF00722	19
	GT34; α-1,6-XylT	PF05637	4	GH31; α-Xylosidases	PF16863, PF01055	4
	GT47; β-1,2-GalT, β-1,2-GalAT	PF03016	5	GH35; β-Galactosidase	PF13364, PF02140, PF01301	1
	GT37; α-1,2-FucT	PF03254	1	GH95; α-1,2-Fucosidase	PF14498	2
	TBL13, 19, 20, 27; *O*-acetylT	PF14416, PF13839	8			
RG-I	GT106; RG-I α-RhaT (RRT)	PF10250	15	PL4; RG-I endolyase	PF14686, PF14683, PF06045	8
	GT116; RG-I α-1,2-GalAT(RGGAT)	PF04765	3	GH35; β-Galactosidase	PF13364, PF02140, PF01301	16
	GT116; α-1,2-GalAT	PF04765	7			
	GT92; β-1,4-GalT (GALS)	PF13704	2			
	GT92; β-1,4-GalT, α-AraT	PF13704	4			
	TBL10, 16; *O*-acetylT	PF14416, PF13839	7			
	GT47; α-1,5-AraT	PF03016	12			
HG	GT8; α-1,4-GalAT	PF01501	24	PL3; Polysacchlyase	PF12708	30
	GT47; β-1,4-XylT	PF03016	6	PLL; Pectate lyase-like	PF00544	21
	PMT; Pectin MethylT	PF03141	2	GH28; Polygalacturonase	PF00295	13
	TBL2, 12, 38, 42, 43; *O*-acetylT	PF14416, PF13839	11			
	TBL38; *O*-acetylE	PF14416, PF1383	5			
HG and RG-I	PMT; Pectin MethylT	PF03141	32	PME; Pectin methylE	PF04043, PF01095	39
	TBL1, 5–7, 18, 39; *O*-acetylT	PF14416, PF13839	14	PMEI; PME inhibitor	PF04043	25
				PAE; Pectin acetylE	PF03283	13
1,3-Glucan	GT48; Callose synth	PF04652, PF14288, PF02364	14	GH17; 1,3-glucosidase	PF00332	43
Xylan	GT47; β-1,4-XylT, β-1,4-GlcAT	PF03016	6	GH3; Glc/xylosidase	PF14310, PF00933, PF01915	8
	GT43; β-1,4-XylT	PF03360	3	GH51; Xyl/Arabinofuranosidase	PF06964	3
	GT61; β-1,2-O-XylT, β-1,4-XylT, α-1,3-AraT	PF04577	6	GH10; Xylanase	PF00331	3
	GT8; α-GlcAT, GalAT	PF01501	5			
	TBL3, 29, 31, 34, 35; *O*-acetylT	PF14416, PF13839	8			
	RWA; *O*-acetylT	PF07779	3			
Mannan	CSLA, D	PF13632	19	GH5; Endomannanase	PF00150	7
	GT106; Man synth	PF10250	2			
	GT34; α-1,6-GalT, α-1,2-GalT	PF05637	1			
	TBL23, 25, 26	PF14416, PF13839	6			
AGP	GT31; β-1,3-GalT	PF13334, PF01762	18	GH35; β-Galactosidase	PF01301	3
	GT106; Pectic AG synthase	PF10250	2			
	GT29; β-1,6-GalT	PF00777	1			
Cellulose, XyG	Expansins	PF03330, PF01357	23			
Cellulose, HG and RGI	Fasciclin-like AGP	PF02469	17			
Unknown	BURP: PG1β-like, RD22, BNM2-like	PF03181	11			
Unknown	GT29; α-sialylT	PF00777	4			
Unknown	GT106; OFUT	PF10250	30			
Unknown	GT47	PF03016	12			
Unknown	CSLG	PF13632	3			
Unknown	GT8; Galactinol synthase	PF01501	5			
N-glycan	GT31; β-1,3-GalT GT8; GalT	PF13334, PF01762; PF01501	3 2	GH29; α-Fucosidase 1	PF01120	2
Unknown				GH1; β-Glucosidase	PF00232	11
Chitin, Cellulose				GH19; Chitinase (like)	PF00182	7
Unknown				GDSL; acetylE	PF00657	66

## Data Availability

Data are contained within the article and Supplementary Materials. The RNA-seq data have been deposited at the European Nucleotide Archive (http://www.ebi.ac.uk/ena/, accessed on 20 August 2024) under the accession number PRJNA884180.

## Supplementary Materials

The following supporting information can be downloaded at https://www.mdpi.com/article/10.3390/ijms26020738/s1, References [23,24,25,27,28,29,30,31,32,33,34,35,108,109,110] are cited in Supplementary Materials.
