# Supplementary material for: A Fresh Look at Celery Collenchyma and Parenchyma Cell Walls Through a Combination of Biochemical, Histochemical, and Transcriptomic Analyses
Source: Int J Mol Sci. 2025 Jan 16;26(2):738. doi: 10.3390/ijms26020738 (PMC11765706; doi:10.3390/ijms26020738)
Supplement: Supplementary file 1 [file ijms-26-00738-s001.zip › Figures S1-S6_merged.pdf]

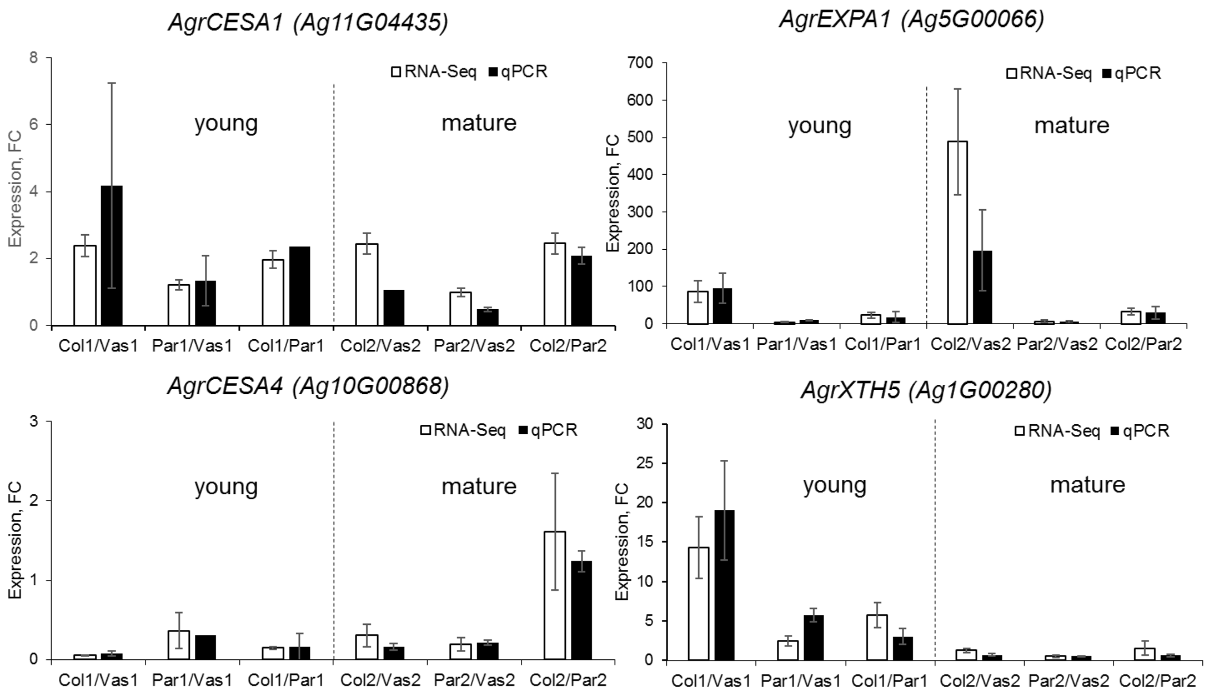

Gene Name	Gene ID	AT ortholog	Primer Sequence 5'-3'	Gene description
AgrEF_TU	*Ag9G01513*	*AT5G10630*	F: GCGGTACTGGAGGTGGTTAC	Elongation factor Tu
			R: ACCCTTCCCAGAGGCTTACT	
AgrCESA1	*Ag11G04435*	*AT4G32410*	F: AGTTGGTTCGGATTCGCCAT	Cellulose synthase A1
			R: AATCCTTTAGGGGCTTGGGC	
AgrCESA4	*Ag10G00868*	*AT5G44030*	F: CTTACCTCGATCGCCTGTCC	Cellulose synthase A4
			R:GCACCCACCTCCTAGCAAAT	
AgrXTH5	*Ag1G00280*	*AT5G13870*	F: GAAGACGGACTGGTCCAAGG	Xyloglucan endotransglucosylase/hydrolase 5
			R: TTCACTGACGACTCGCATCC	
AgrEXP10	*Ag5G00066*	*AT1G26770*	F: GCACTGAGCACAGCATTGTT	Expansin A10
			R: GTGATTGTACCACGGAGGCA	

Figure S1. A comparison of gene expression levels determined by RNA-Seq and qRT-PCR. Error bars show the standard error of the mean (n=3-4). The gene list and primer sequences are given in the table. Col – collenchyma, Par – parenchyma, Vas – vascular bundle.

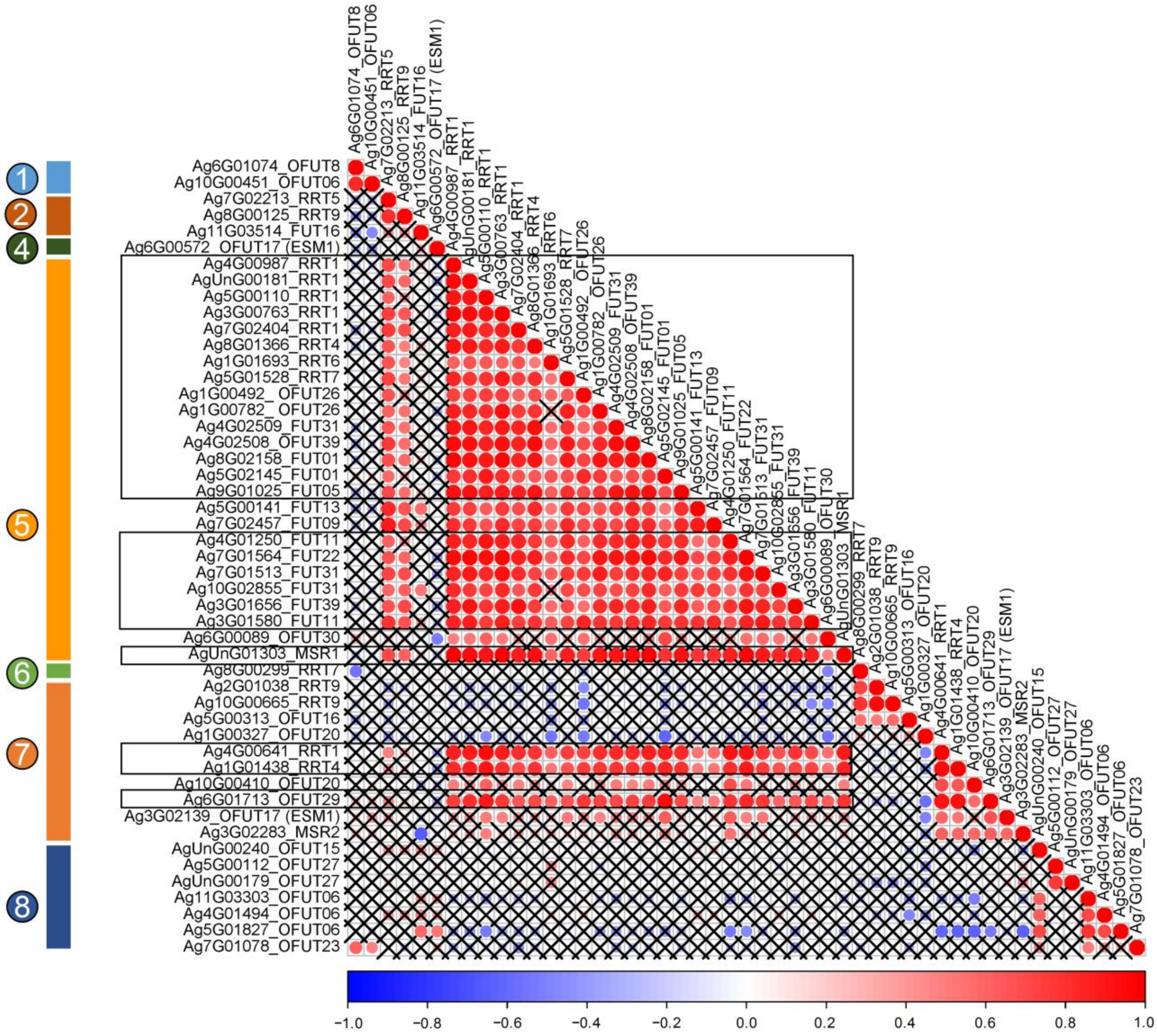

Figure S2. Correlation of expression of *GT106* genes in celery petiole tissues. Genes that are positively coexpressed with others are marked by red dots, while those that are negatively coexpressed are marked by blue dots. The size and color of the dots reflect the absolute value of Pearson's correlation coefficient (p<0.01; insignificant p-values are marked with X, Table S5). Black frames indicate coexpressed genes that were upregulated in young collenchyma, and this set was used for further coexpression analysis presented in Figure 9. On the left, the color panel and circles correspond to the clustering of genes for cell wall-related proteins presented in Figure 7.

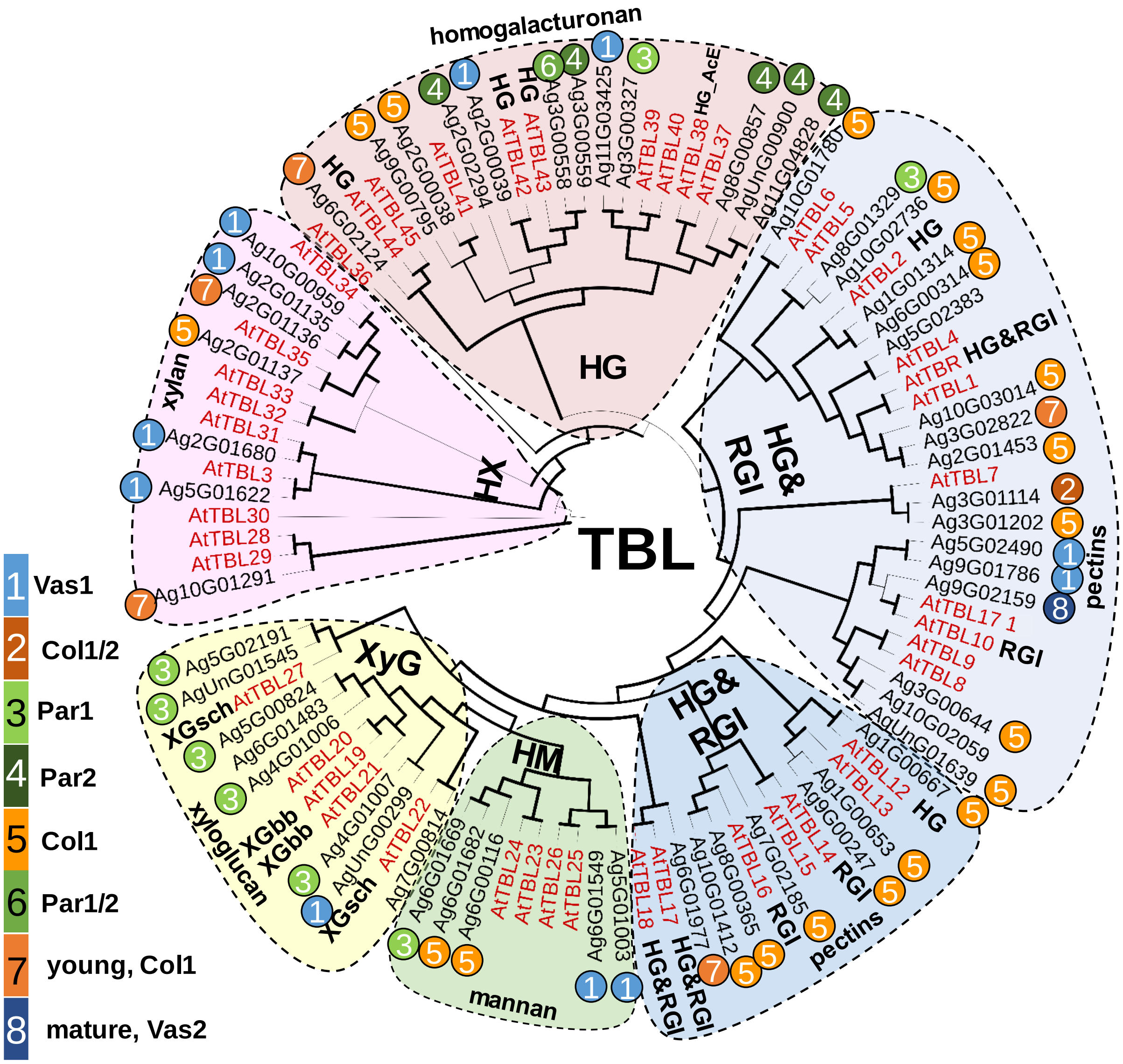

Figure S3. Phylogeny of celery (black) and Arabidopsis (red) Trichome Birefringence-Like (TBLs) proteins. The model of substitution Q.yeast+R6, ultrafast bootstrap support 10,000. Branches with maximum bootstrap values have the maximum width. Cluster numbers, as well as known substrate specificity and activity (UniProt; [64]), are indicated in circles near the appropriate clade or gene. Polysaccharides: XyG – xyloglucan, RGI – rhamnogalacturonan I, HM – heteromannan, HX – heteroxylan, and HG – homogalacturonan. AcE – acetylesterase activity [64]. Col – collenchyma, Par – parenchyma, Vas – vascular bundles. XyGbb – acetylation of XyG backbone, XyGsch – acetylation of XyG side chains [64]. On the left, the color panel and circles correspond to the clustering of genes for cell wall-related proteins presented in Figure 7.

References are numbered in accordance with the main text.

64. Dauphin, B.G.; Ropartz, D.; Ranocha, P.; Rouffle, M.; Carton, C.; Le Ru, A.; Martinez, Y.; Fourquaux, I.; Ollivier, S.; Mac-Bear, J.; Trezel, P.; Geairon, A.; Jamet, E.; Dunand, C.; Pelloux, J.; Ralet, M.-C.; Burlat, V. TBL38 Atypical Homogalacturonan-Acetylesterase Activity and Cell Wall Microdomain Localization in Arabidopsis Seed Mucilage Secretory Cells. *iScience* **2024**, *27*(5), 109666. https://doi.org/10.1016/j.isci.2024.109666.

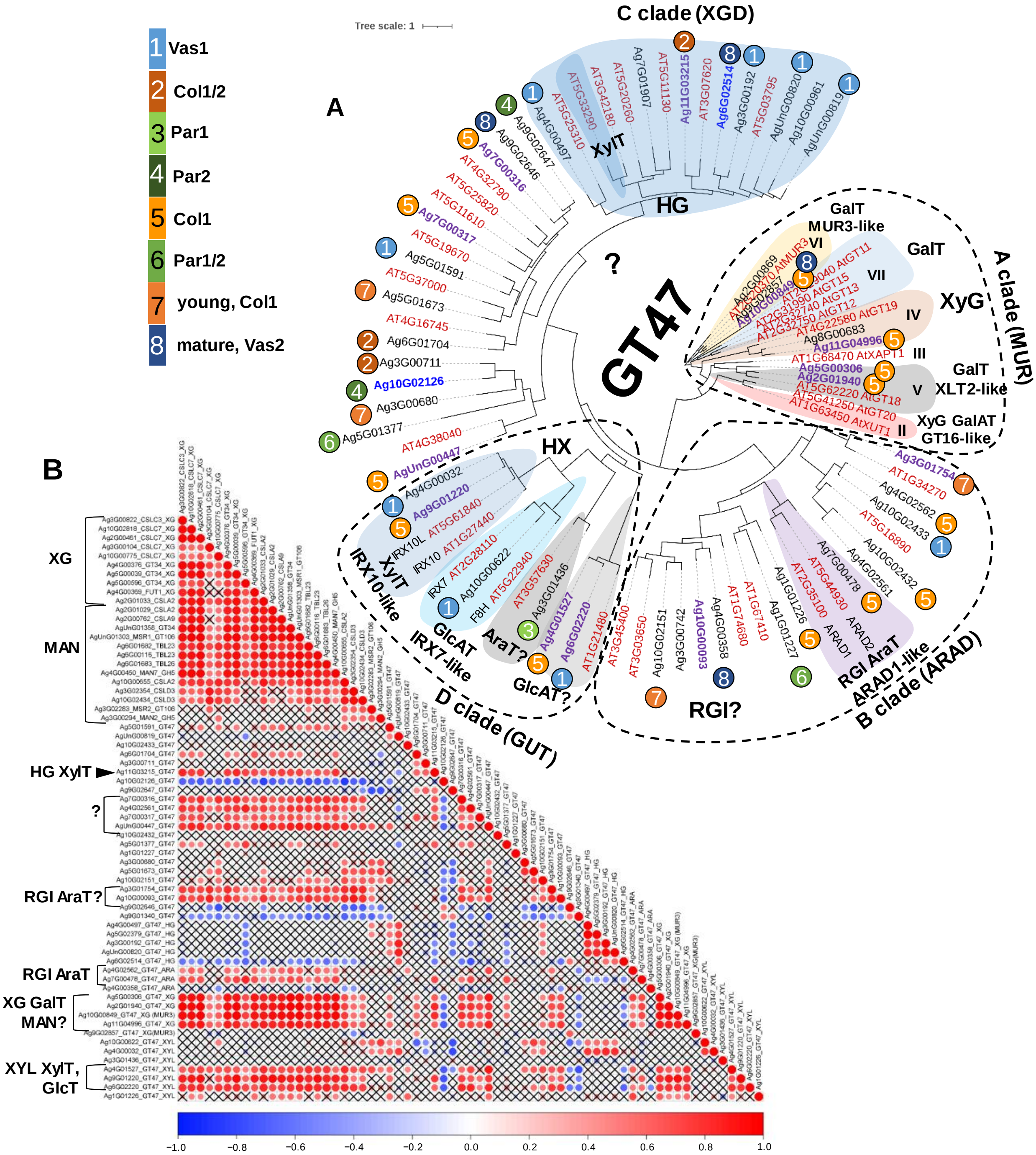

Figure S4. (**A**) Phylogeny of celery (black) and Arabidopsis (red) glycosyltransferase 47 family (GT47) protein sequences. The model of substitution WAG+F+I+R6, ultrafast bootstrap support 10,000. Known substrate specificity and activity are indicated in circles near the appropriate clade. The clade annotation was based on [70]. Violet or blue fonts on the tree denote genes that significantly positively or negatively coexpressed with genes for XyG and mannan-related proteins, respectively (B). Cluster numbers are designated near the appropriate gene. Polysaccharides: XyG – xyloglucan, RGI – rhamnogalacturonan I, HM – heteromannan, HX – heteroxylan, and HG – homogalacturonan. GT activities: GalT –galactosyltransferase, GalAT – galacturonosyltransferase, AraT – arabinosylransferase, XylT – xylosyltransferase, and GlcAT – glucuronosyltransferase. Xylosyltransferase activity was experimentally confirmed for AT5G33290 and predicted for other sequences in the clade [110]. (**B**) Correlation of expression of GT47 genes with genes encoding enzymes involved in xyloglucan (XyG) and heteromannan (HM) biosynthesis. Genes that are positively coexpressed with others are marked by red dots, while those that are negatively coexpressed are marked by blue dots. The size and color of the dots reflect the absolute value of Pearson's correlation coefficient (p<0.01; insignificant p-values are marked with X, Table S5). The color panel and circles correspond to the clustering of genes for cell wall-related proteins presented in Figure 7. Col – collenchyma, Par – parenchyma, Vas – vascular bundle.

References are numbered in accordance with the main text.

70. Mikkelsen, M.D.; Harholt, J.; Ulvskov, P.; Johansen, I. E.; Fangel J.U; Doblin, M.S.; Bacic, A.; Willats, W.G.T. Evidence for Land Plant Cell Wall Biosynthetic Mechanisms in Charophyte Green Algae. *Ann Bot.* **2014**, *114* (6), 1217–1236. https://doi.org/10.1093/aob/mcu171.

110. Jensen, J.K.; Sørensen, S.O.; Harholt, J.; Geshi, N.; Sakuragi, Y.; Møller, I.; Zandleven, J.; Bernal, A.J.; Jensen, N.B.; Sørensen, C.; Pauly, M.; Beldman, G.; Willats, W.G.T.; Scheller, H.V. Identification of a Xylogalacturonan Xylosyltransferase Involved in Pectin Biosynthesis in Arabidopsis. *Plant Cell* **2008**, *20*(5), 1289–1302. https://doi.org/10.1105/tpc.107.050906.

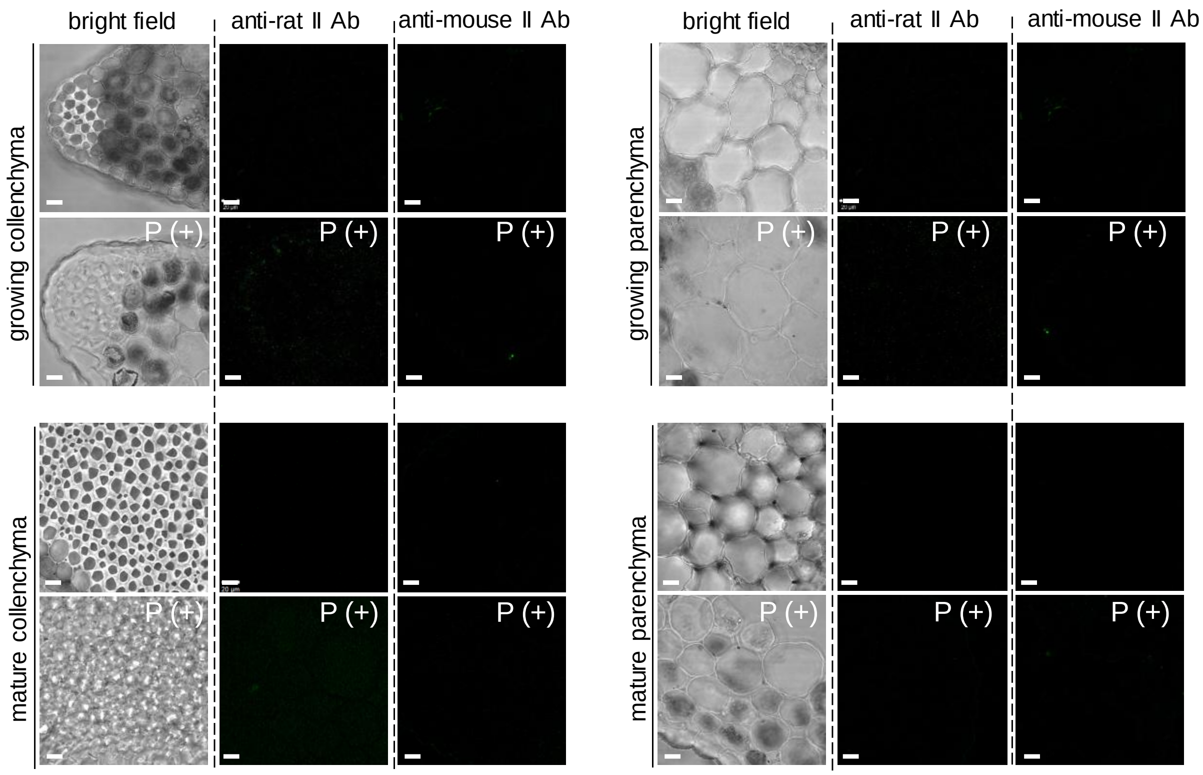

Figure S5. Cross-sections used as negative controls (the primary antibody was not added during immunolabeling). P (+) – sections after pectinase pretreatment. Bright field images display the morphology of the cells. Scale bar: 20 µm.

Figure S6. Immunolocalization of polysaccharide epitopes in collenchyma and parenchyma cell walls at two developmental stages in 4 replicates.

Long fragments of unesterified homogalacturonan

PAM1

Growing collenchyma

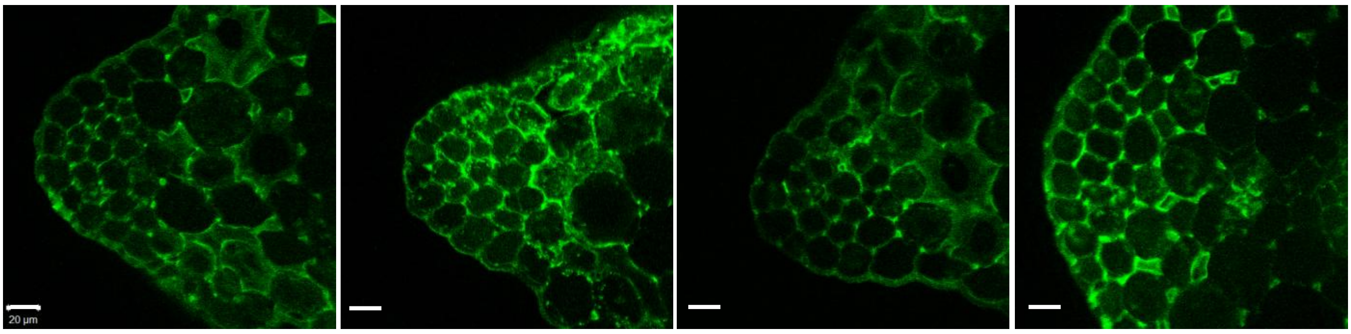

Growing parenchyma

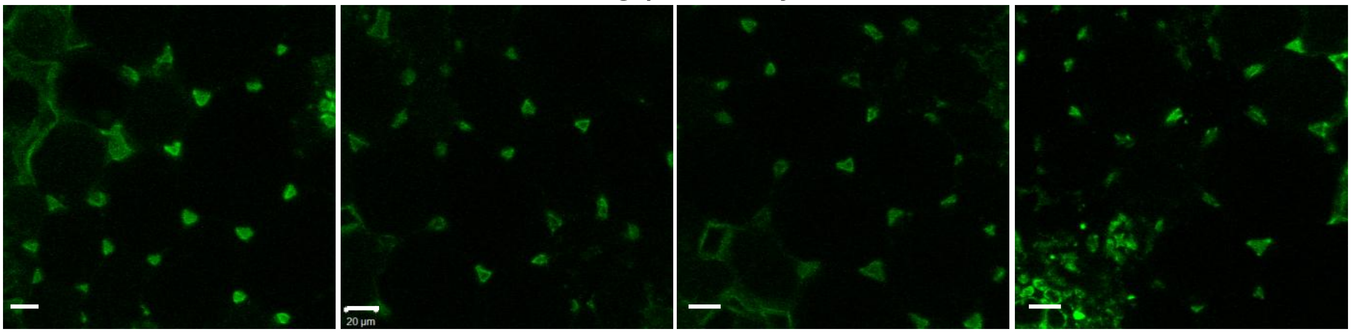

Mature collenchyma

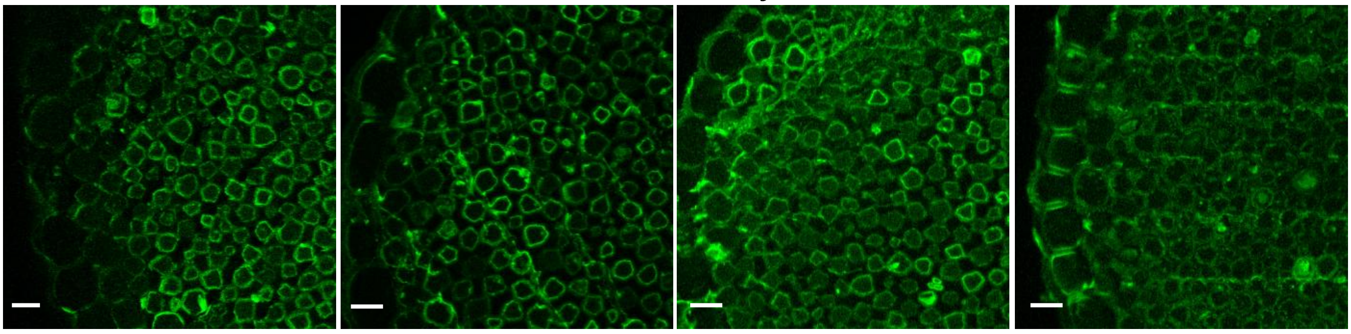

Mature parenchyma

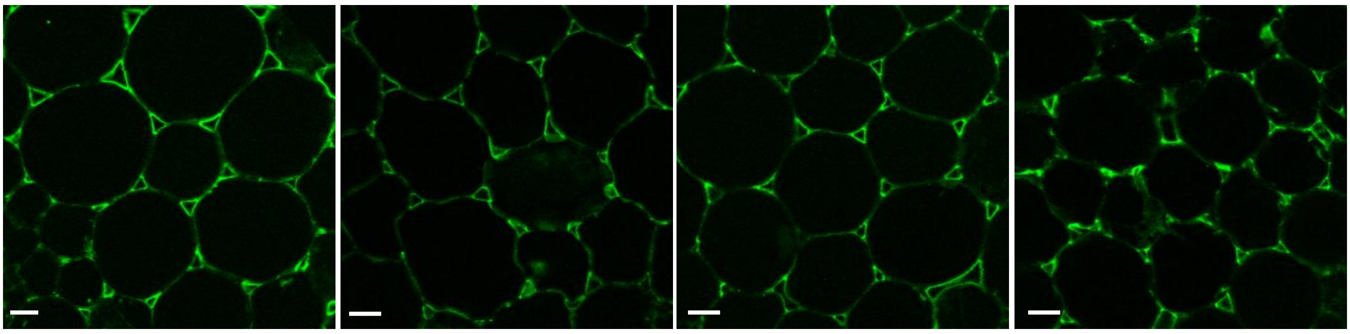

Scale bar 20 µm

Partially esterified homogalacturonan
JIM5

Growing collenchyma

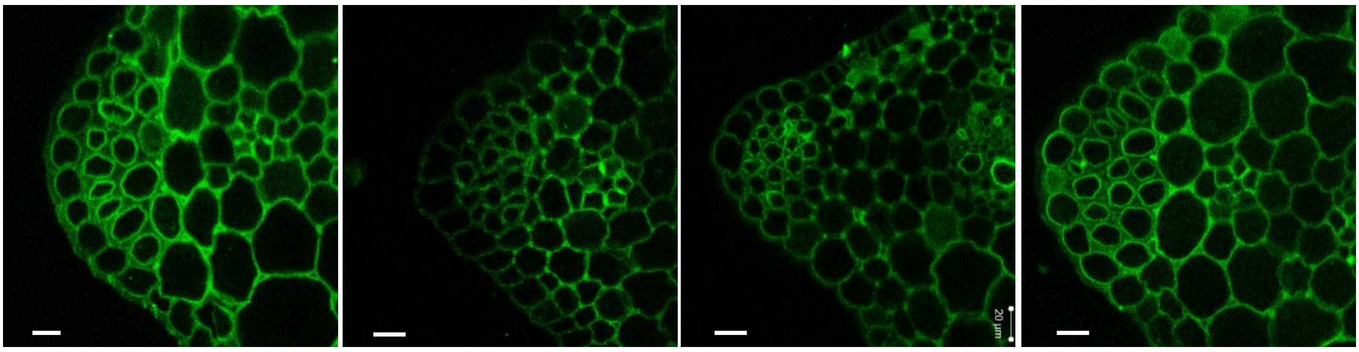

Growing parenchyma

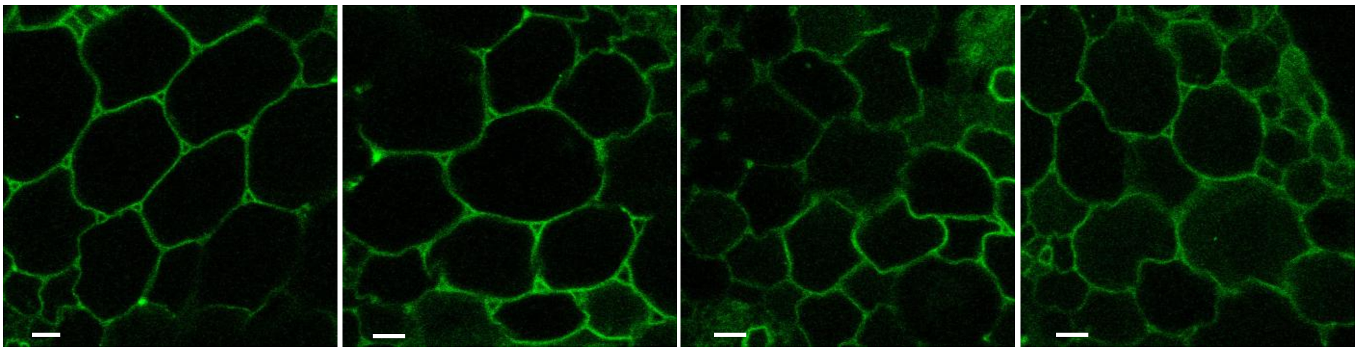

Mature collenchyma

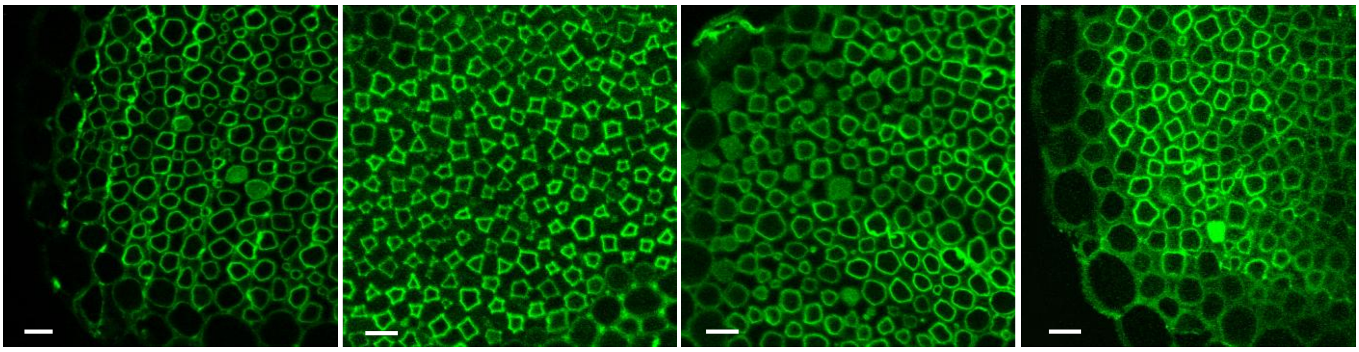

Mature parenchyma

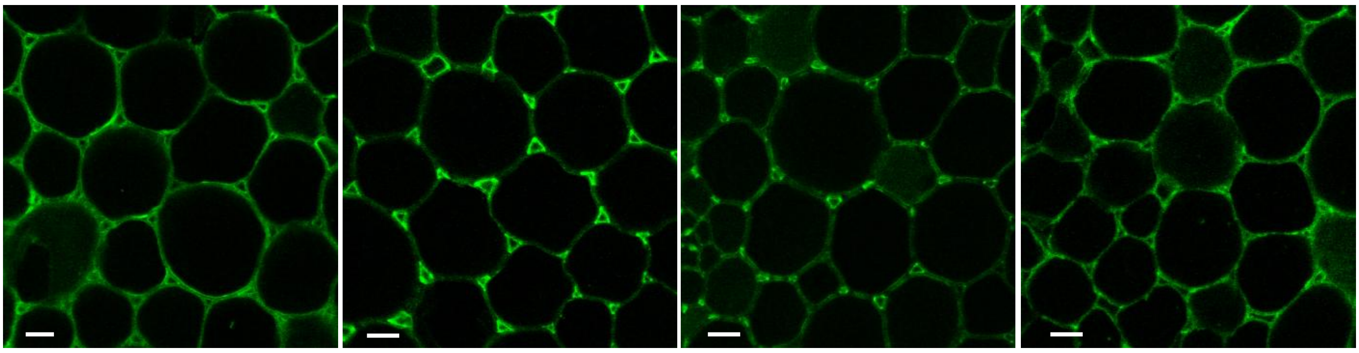

Scale bar 20 µm

Esterified homogalacturonan
JIM7

Growing collenchyma

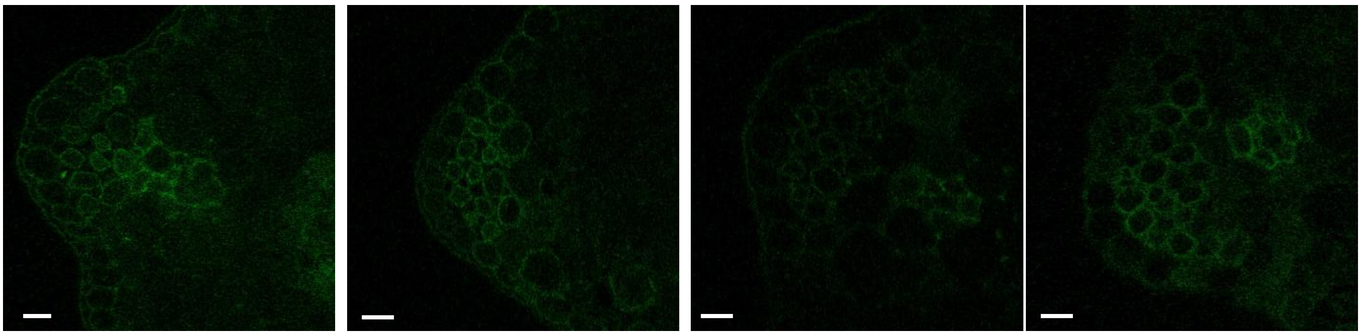

Growing parenchyma

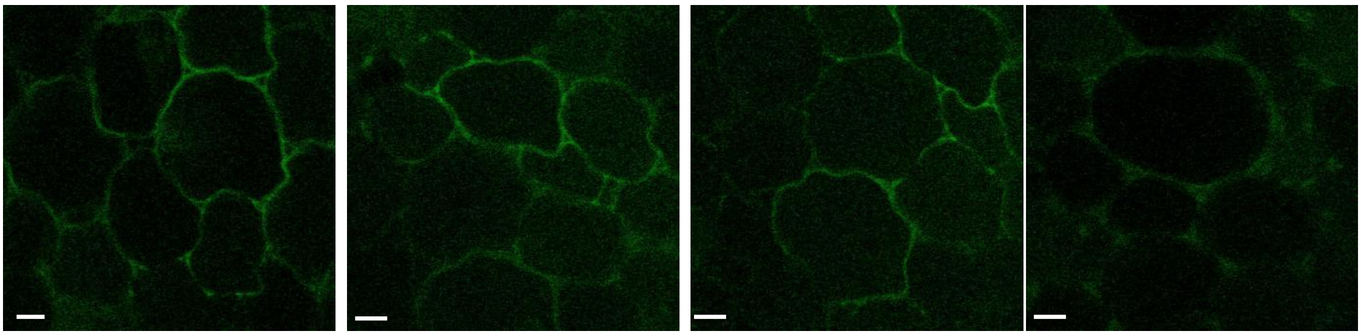

Mature collenchyma

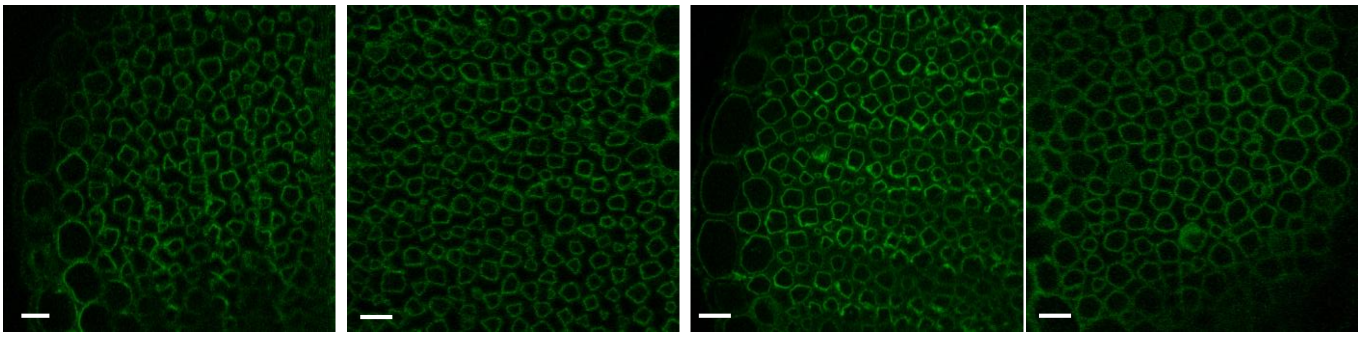

Mature parenchyma

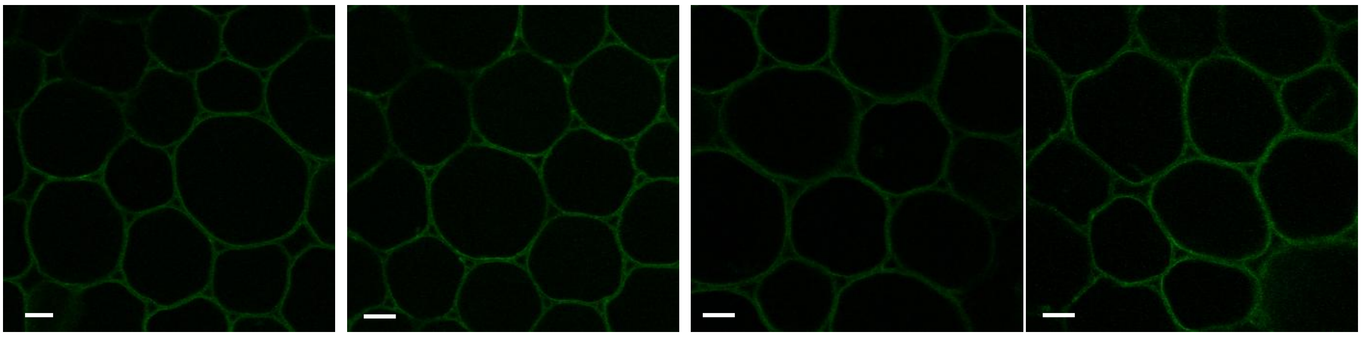

Scale bar 20 µm

Rhamnogalacturonan I backbone
INRA-RU2

Growing collenchyma

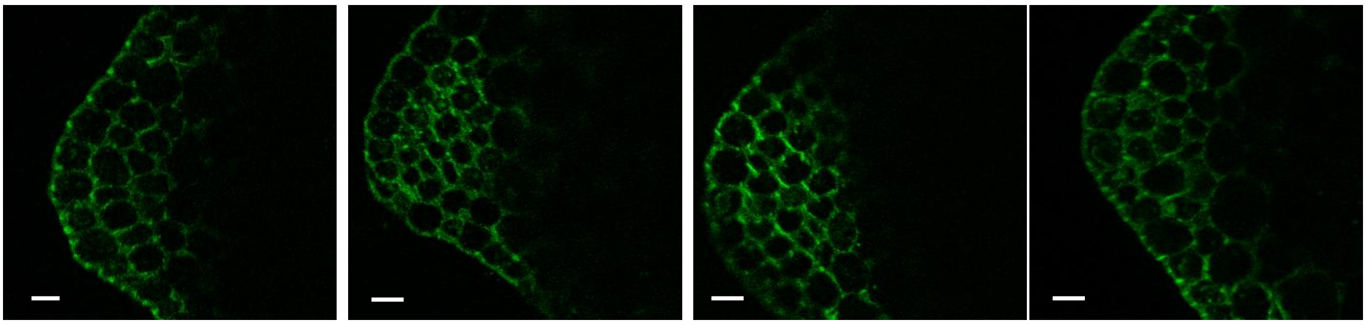

Growing parenchyma

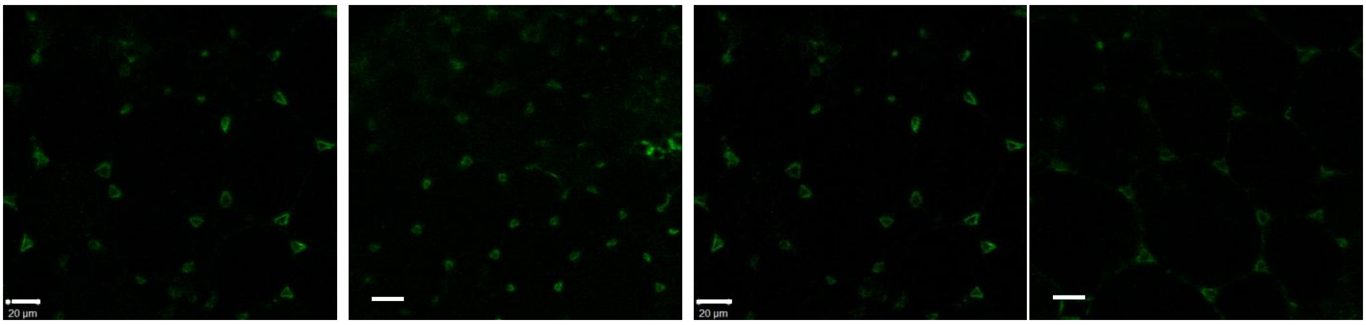

Mature collenchyma

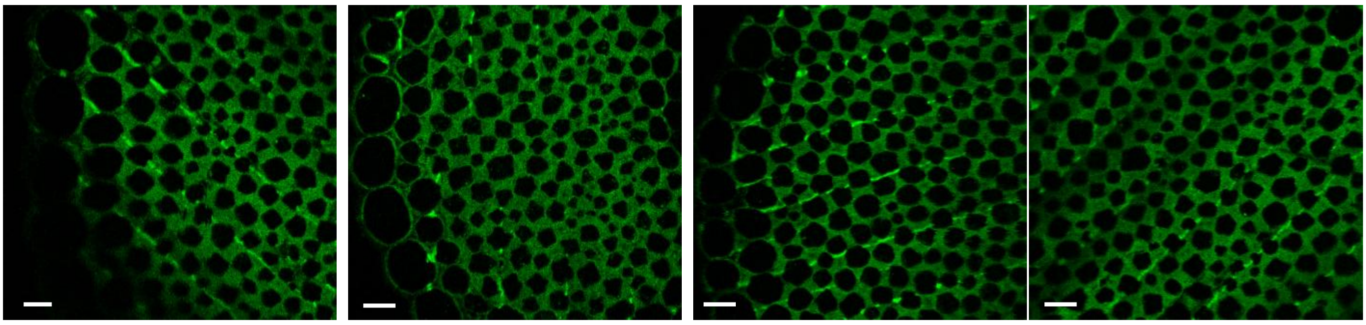

Mature parenchyma

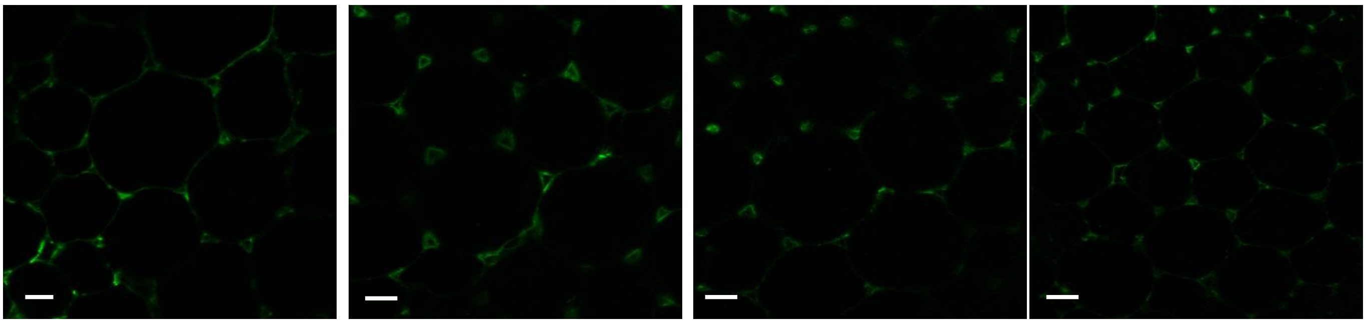

Scale bar 20 µm

Linear β-(1,4)-D-galactan

LM5

Growing collenchyma

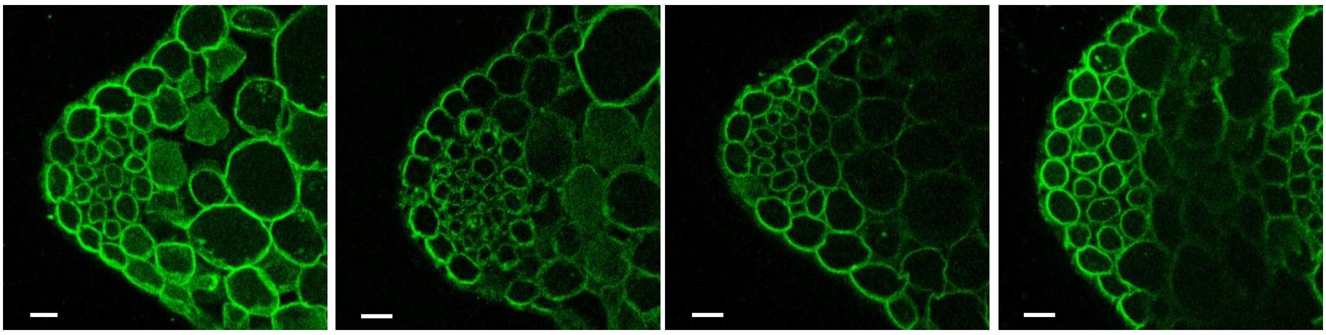

Growing parenchyma

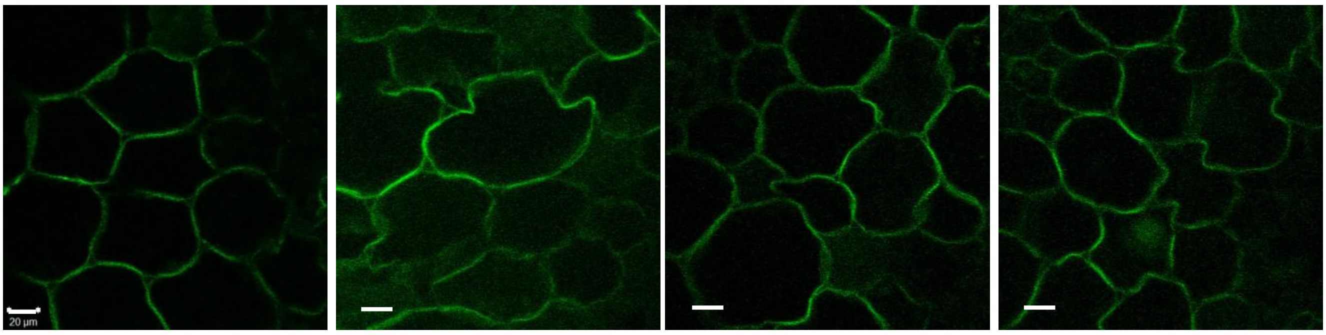

Mature collenchyma

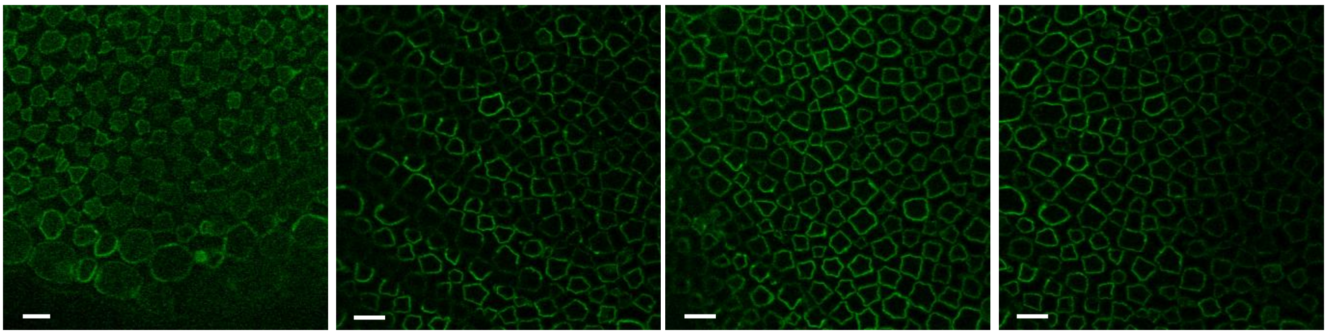

Mature parenchyma

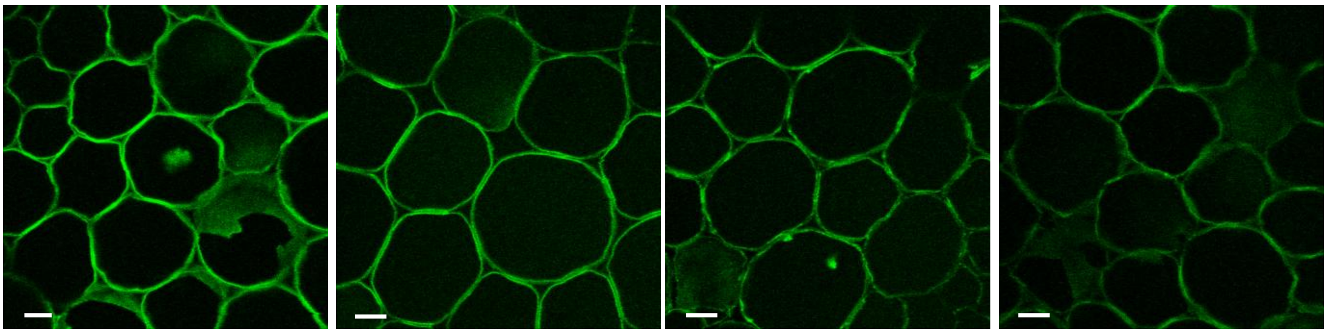

Scale bar 20 µm

Branched β-(1,4)-D-galactan

LM26

Growing collenchyma

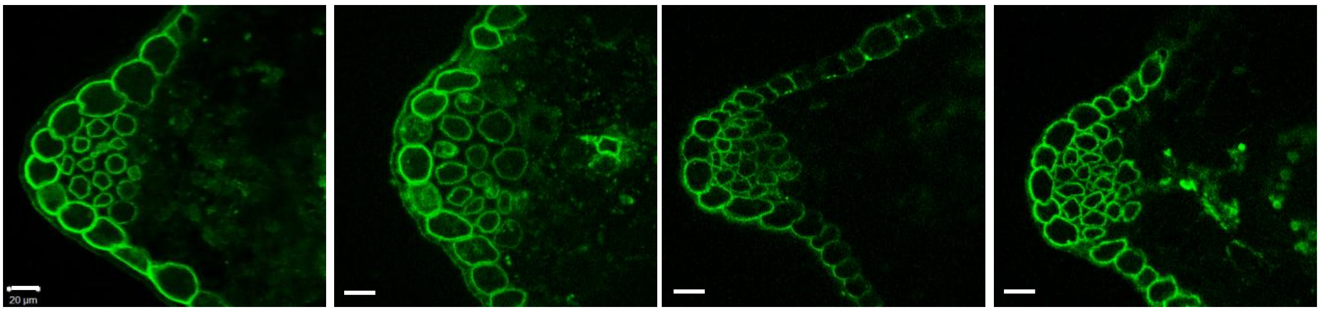

Growing parenchyma

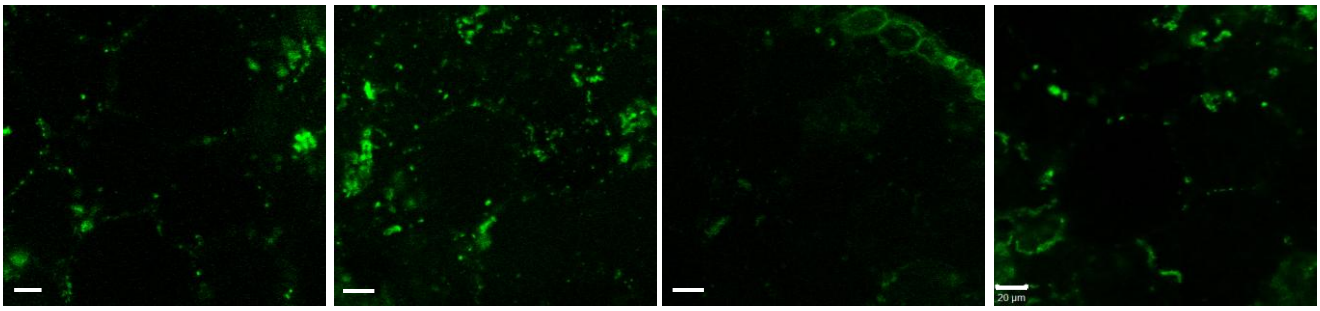

Mature collenchyma

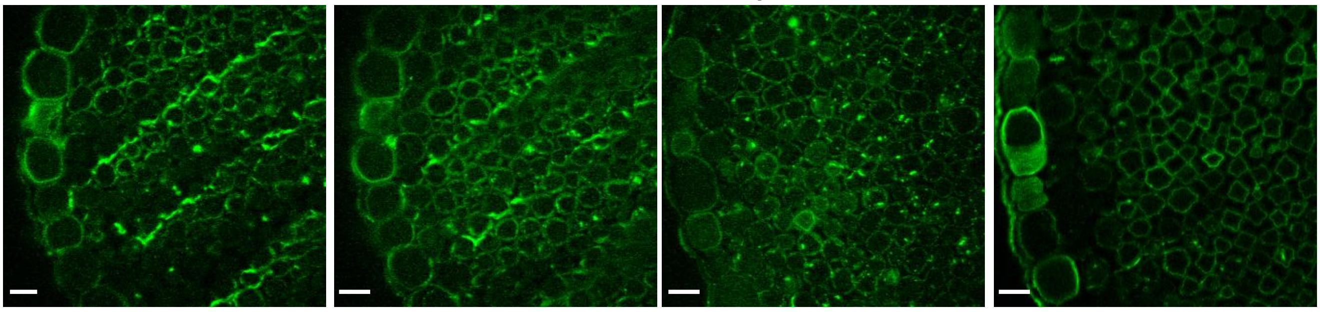

Mature parenchyma

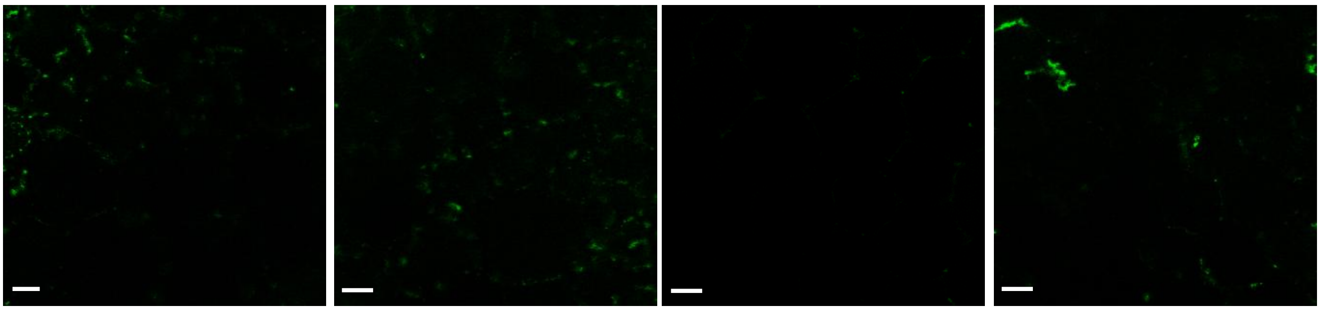

Scale bar 20 µm

Linear α-(1,5)-L-arabinan

LM6

Growing collenchyma

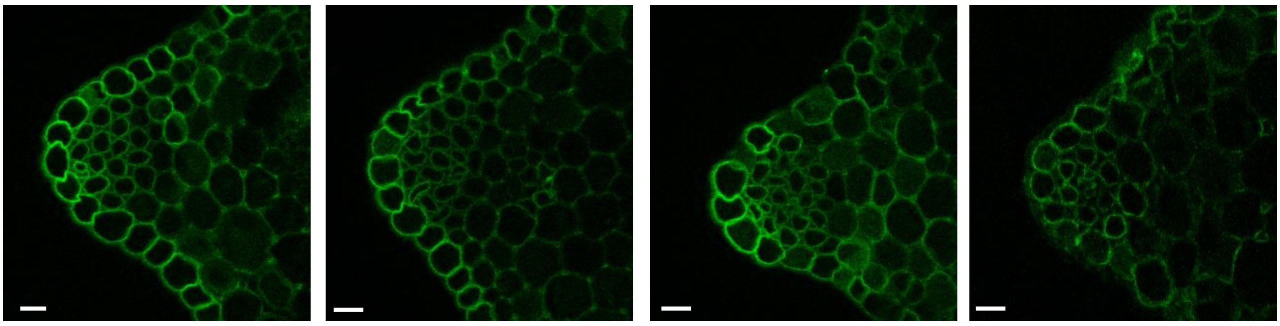

Growing parenchyma

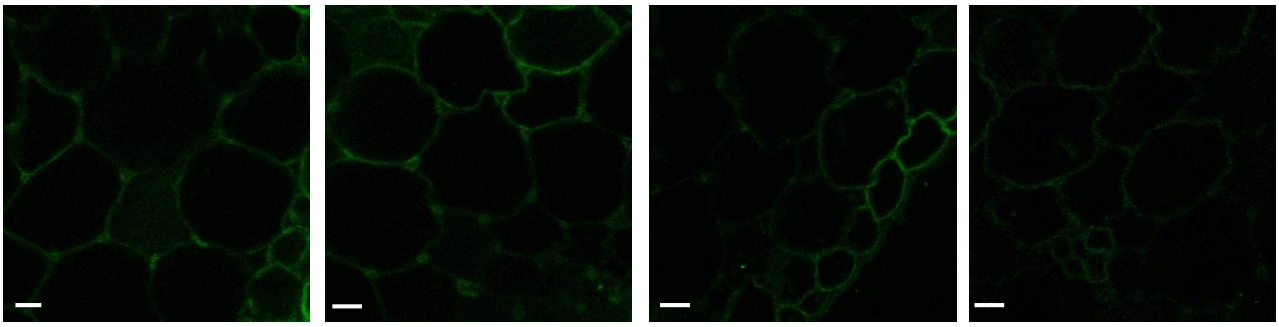

Mature collenchyma

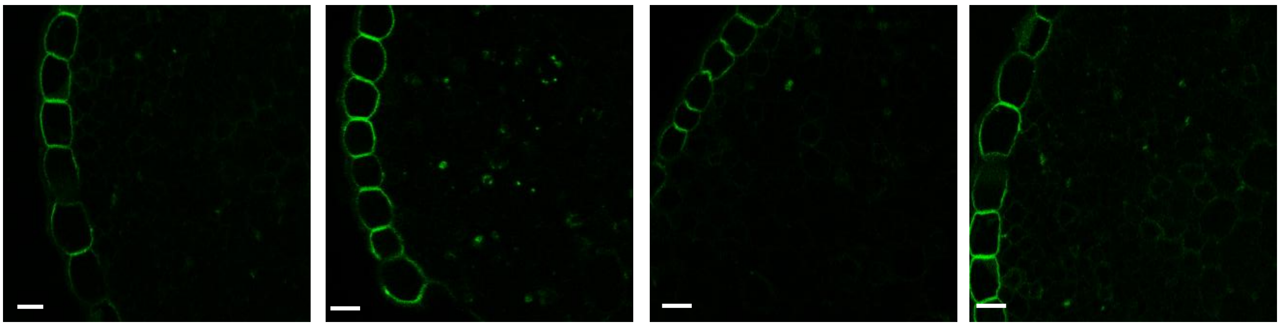

Mature parenchyma

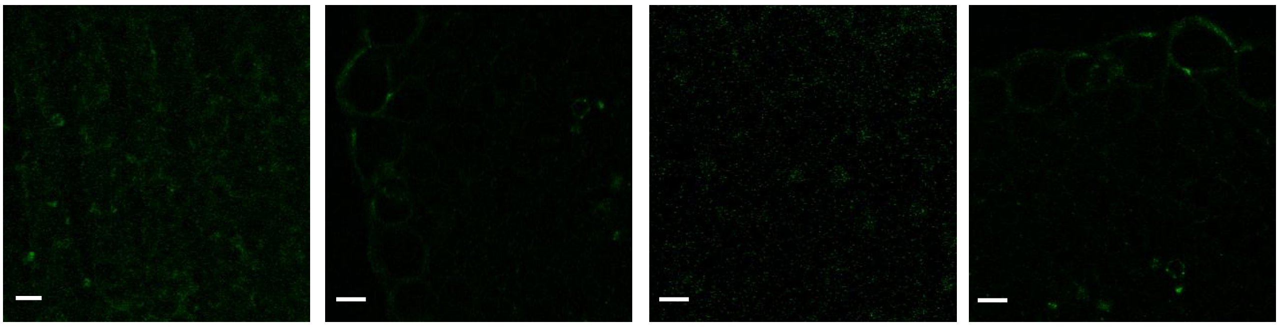

Scale bar 20 µm

Type I arabinogalactan

INRA-AGI-1

Growing collenchyma

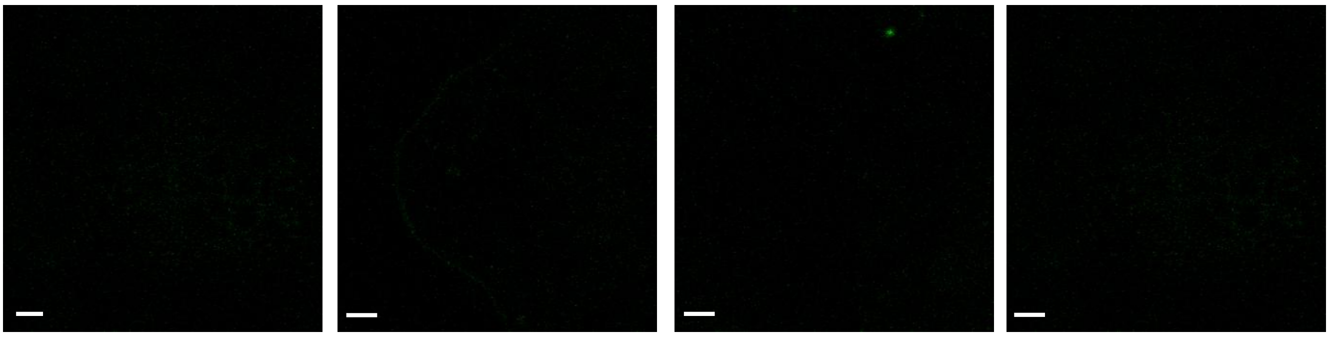

Growing parenchyma

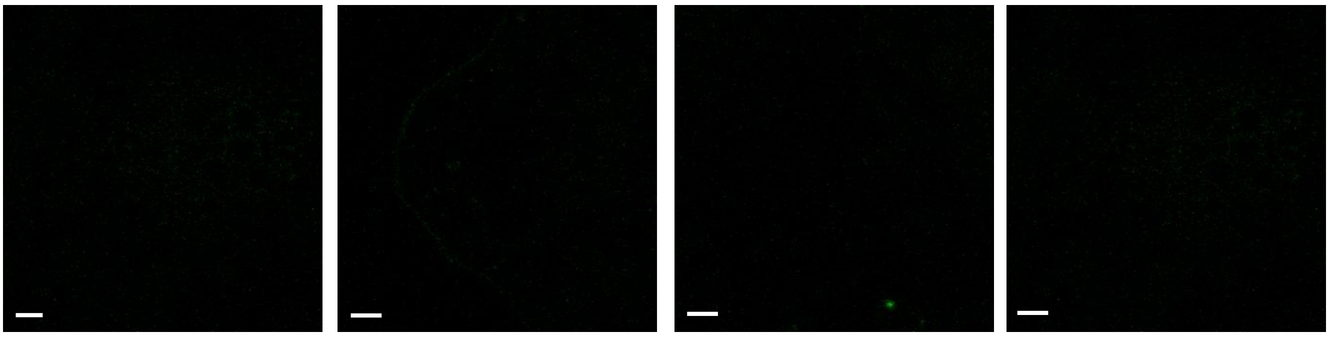

Mature collenchyma

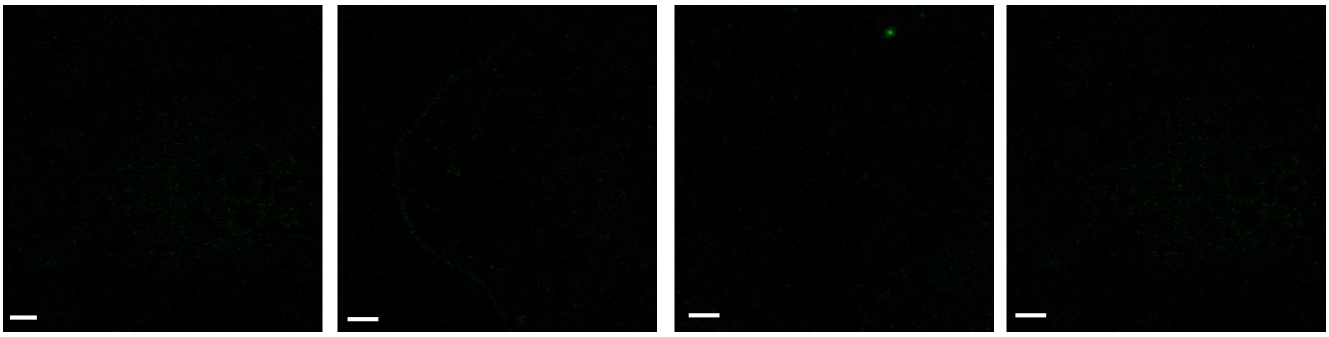

Mature parenchyma

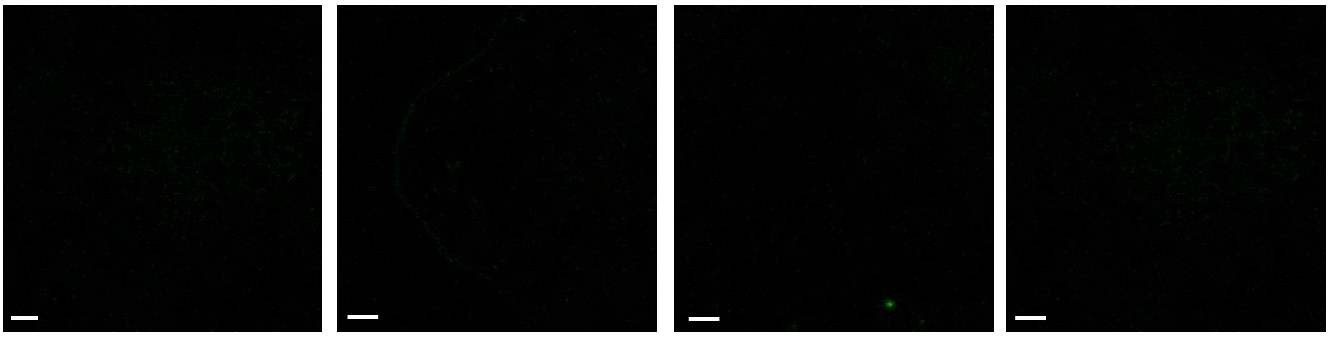

Scale bar 20 µm

XXXG/XXLG/XLLG-motives of xyloglucan

LM25

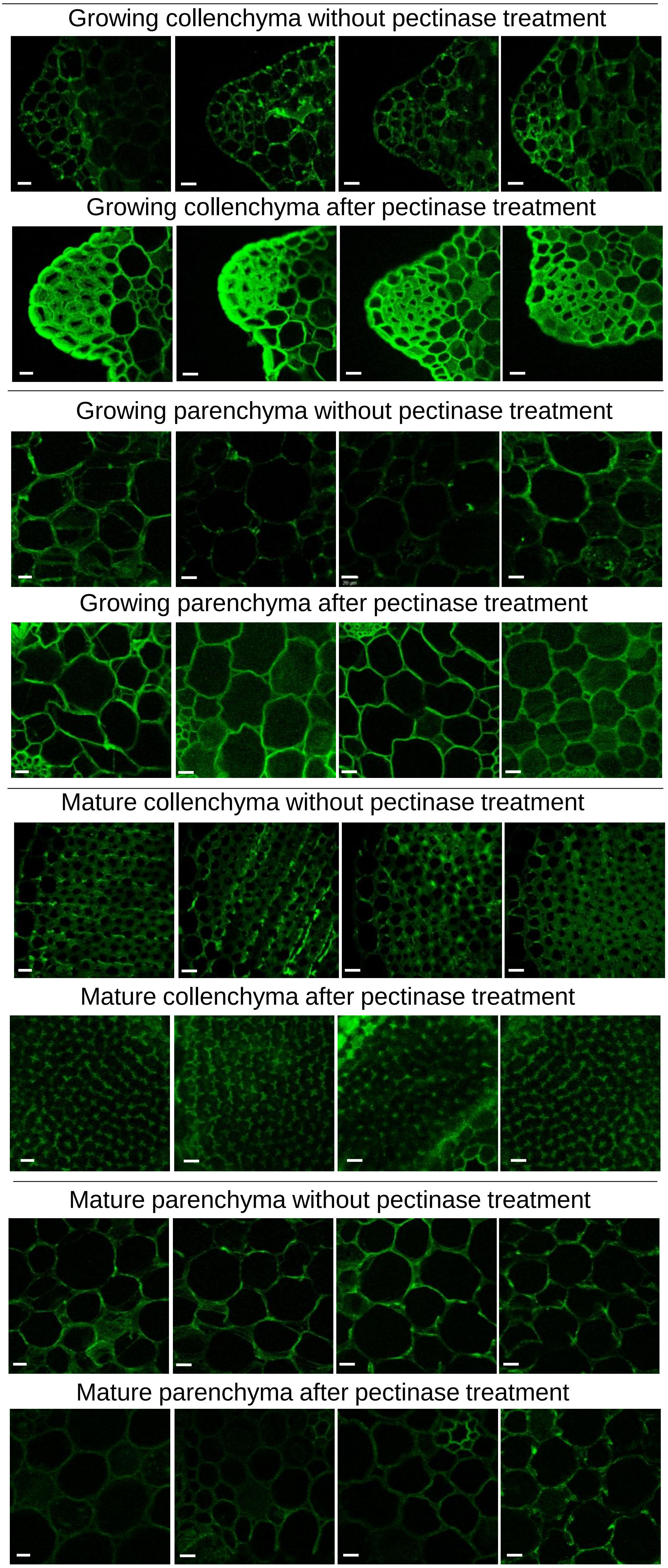

Scale bar 20 µm

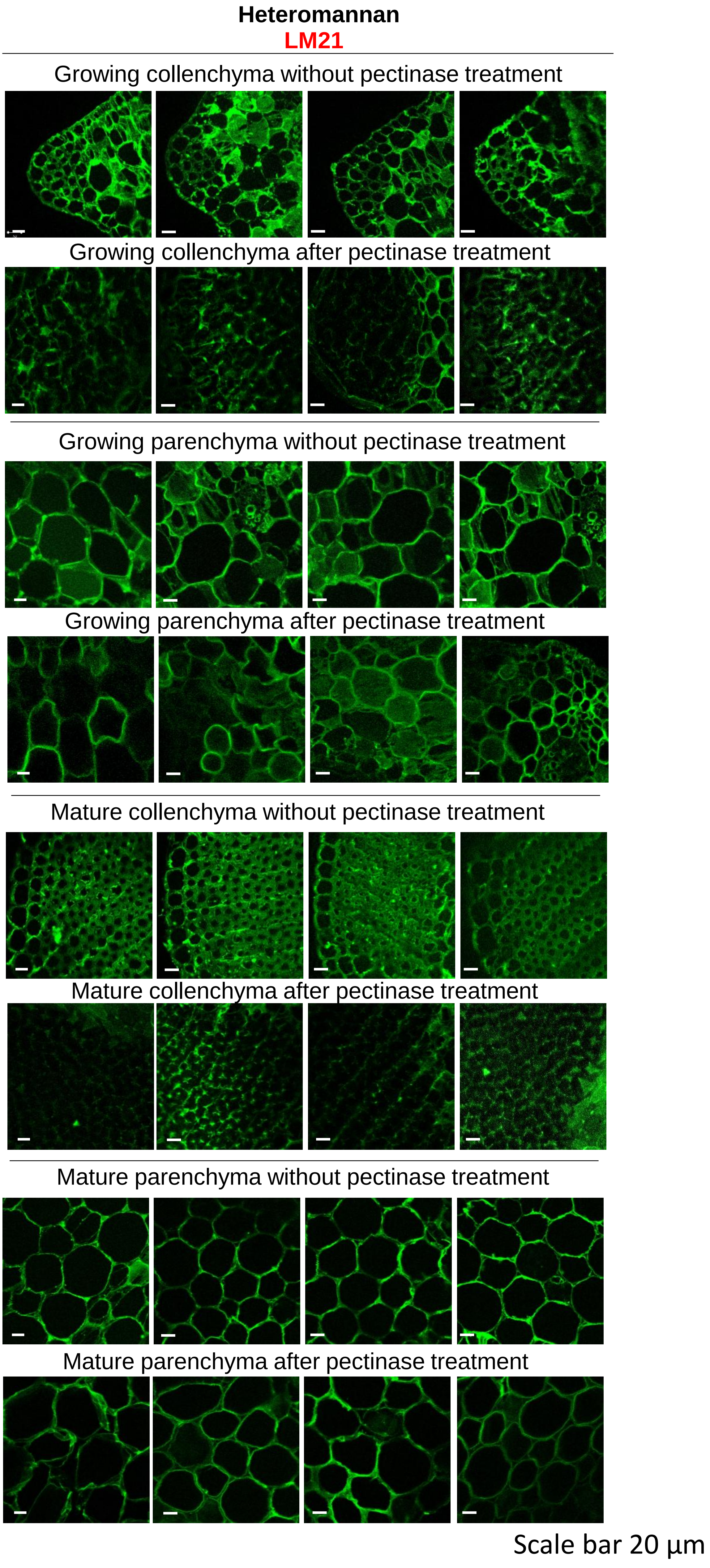
Heteromannan
LM21
Growing collenchyma without pectinase treatment
Growing collenchyma after pectinase treatment
Growing parenchyma without pectinase treatment
Growing parenchyma after pectinase treatment
Mature collenchyma without pectinase treatment
Mature collenchyma after pectinase treatment
Mature parenchyma without pectinase treatment
Mature parenchyma after pectinase treatment
Scale bar 20 µm

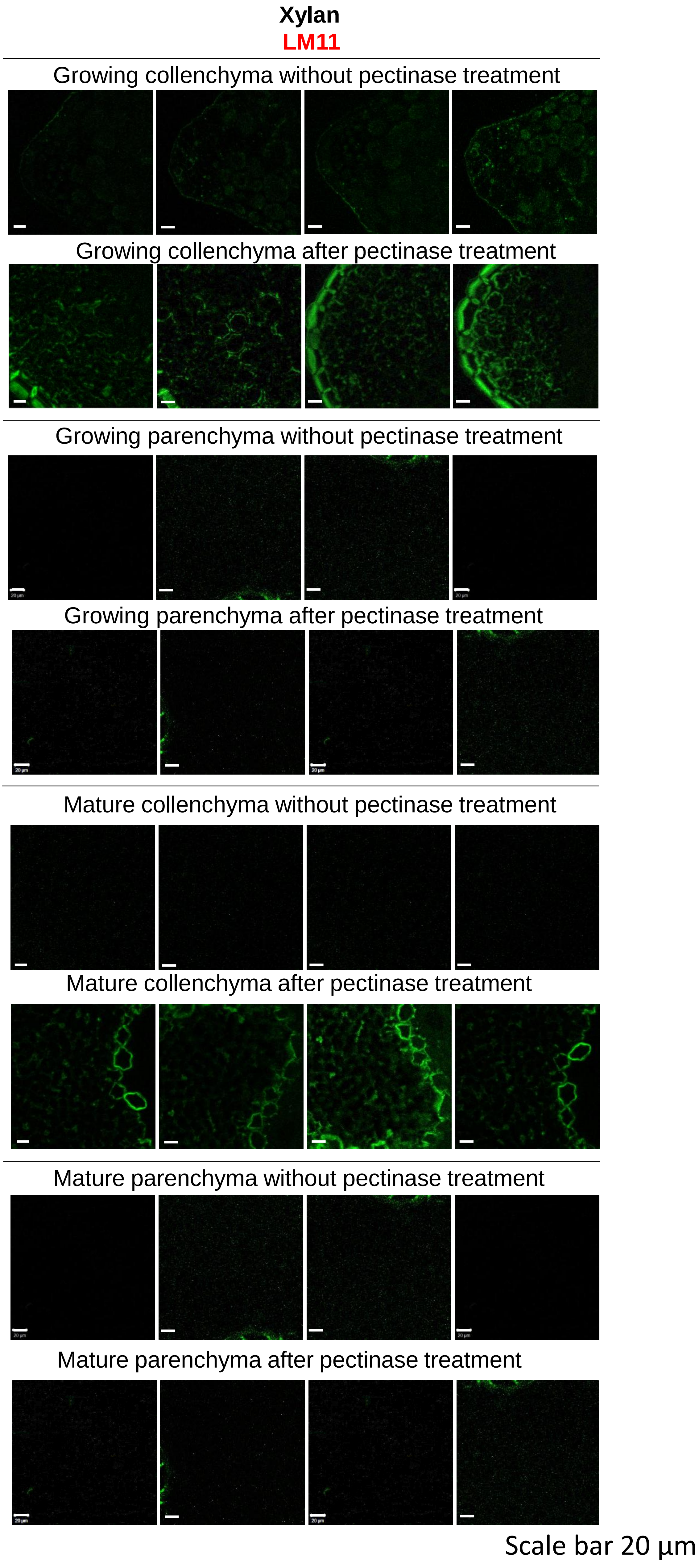
Xylan
LM11
Growing collenchyma without pectinase treatment
Growing collenchyma after pectinase treatment
Growing parenchyma without pectinase treatment
Growing parenchyma after pectinase treatment
Mature collenchyma without pectinase treatment
Mature collenchyma after pectinase treatment
Mature parenchyma without pectinase treatment
Mature parenchyma after pectinase treatment
Scale bar 20 µm

Crystalline cellulose

CBM3a

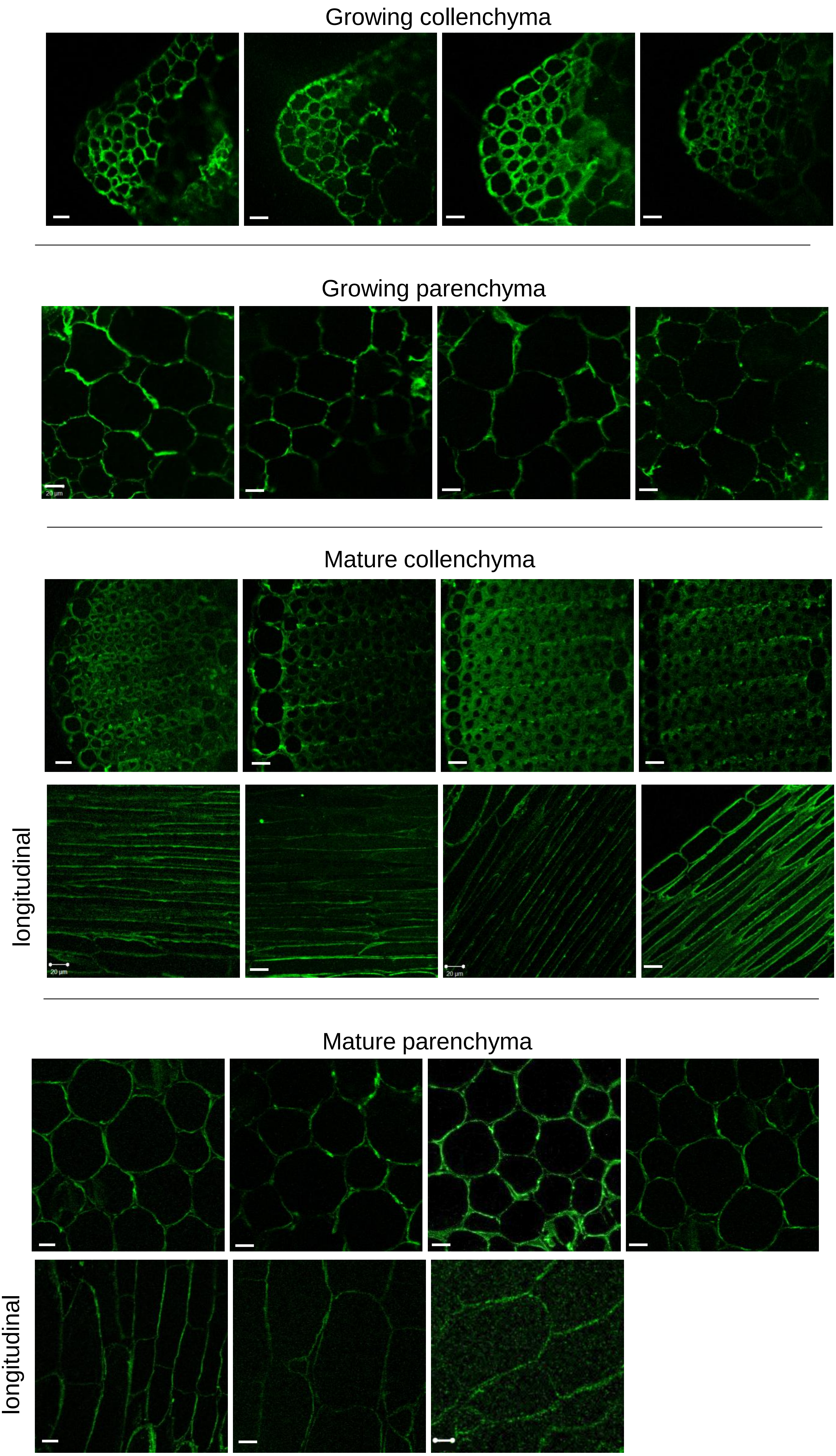

Scale bar 20 µm

Negative controls (no primary antibody added)

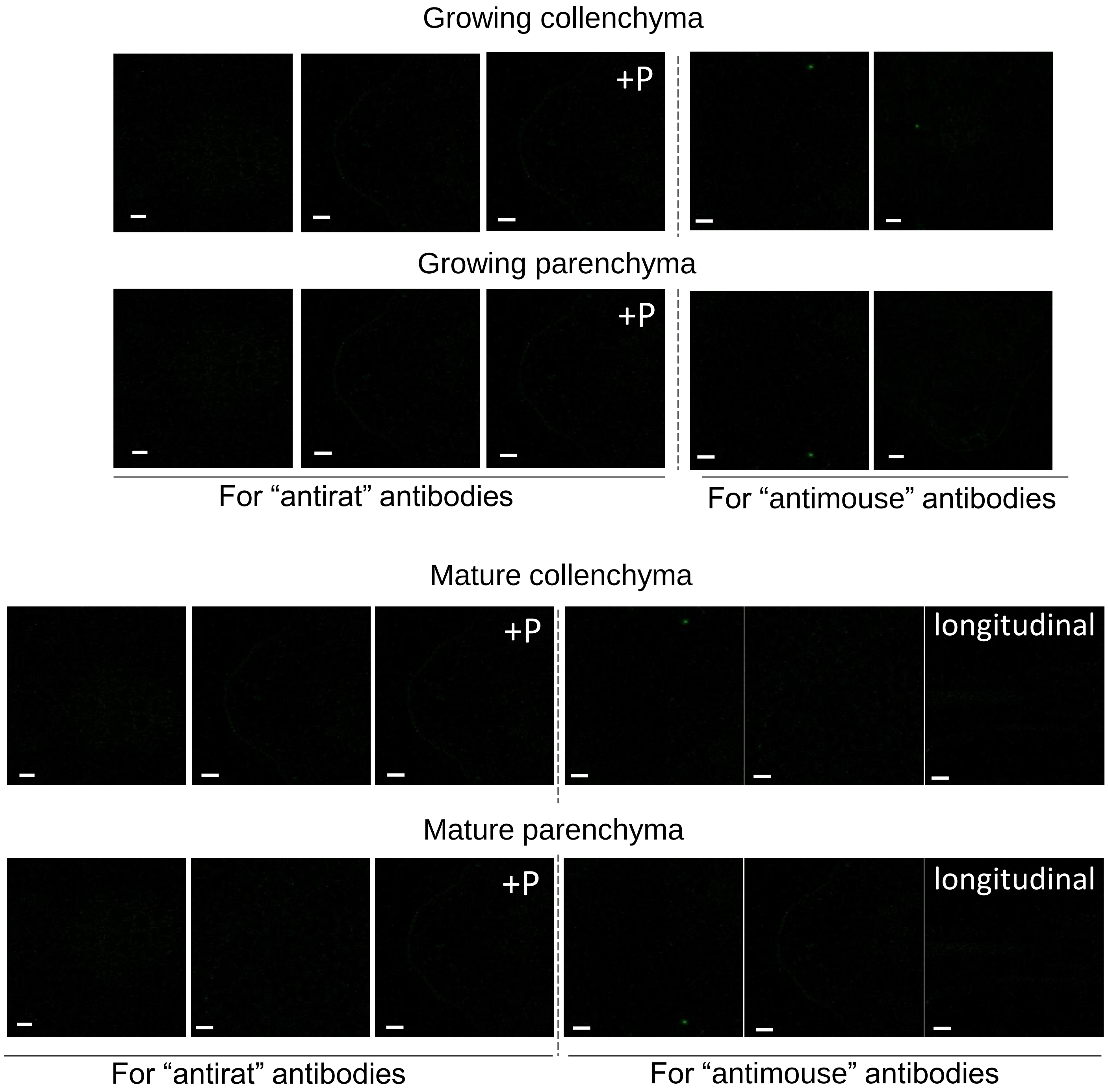

Scale bar 20 µm
