# Supplementary material for: A Fresh Look at Celery Collenchyma and Parenchyma Cell Walls Through a Combination of Biochemical, Histochemical, and Transcriptomic Analyses
Source: Int J Mol Sci. 2025 Jan 16;26(2):738. doi: 10.3390/ijms26020738 (PMC11765706; doi:10.3390/ijms26020738)
Supplement: Supplementary file 1 [file ijms-26-00738-s001.zip › Table S1.pdf]

Table S1. Characteristics of used antibodies.

Antibody	Antigen	Weak labeling	Strong labeling	Ref
PAM1	De-esterified GalA residues	30<OGAs with DPs <34	unesterified GalA, DP >34, low DE	[23, 24]
JIM5	MeGalA-(1,4)-α-GalA-(1,4)-α-GalA-(1,4)-α-GalA-(1,4)-α-GalA-(1,4)-MeGalA	DE>90, DE<20%	methylesterified and non-methylesterified GalA	http://www.carbosource.net, [24]
JIM7	GalA-(1,4)-MeGalA-(1,4)-MeGalA-(1,4)-MeGalA-(1,4)-MeGalA-(1,4)-GalAMeGalA-(1,4)-GalA-(1,4)-MeGalA-(1,4)-GalA-(1,4)-MeGalA-(1,4)-GalA	Low methylesterified GalA	Heavily methylesterified GalA,	http://www.carbosource.net, [24]
INRA-RU2	Rha-(1,4)-GalA-(1,2)-Rha-(1,4)-GalA-(1,2)	DP=4, $Rha_2GalA_2Gal_2$	DP=14-18, substitutions with Gal, Ara, HG do not affect	[25]
LM5	β-Gal-(1,4)-β-Gal-(1,4)-β-Gal-(1,4)-β-Gal	fluorinated and methylated β-(1,4)-Gal_4	β-(1,4)-Gal, DP 3-5	[27,28,108]
LM26	6‴-O-β-(1,6)- Gal-β-(1,4)-Gal_6	6‴-O-β-(1,6)-Gal-β-(1,6)-Gal-β-(1,4)-Gal_6, 6'-O-β-(1,6)-Gal-β-(1,4)-Gal_3	6‴-O-β-Gal-β-(1,4)-Gal_6	[28]
LM6	α-Ara-(1,5)-α-Ara-(1,5)-α-Ara-(1,5)-α-Ara-(1,5)-α-Ara	branched arabinan	α-(1,5)- L-Ara*f*2, linear arabinan	[29,30]
INRA-AGI-1	[(1,4)-β-D-Gal*p*]$_x$-[(1,5)-α-L-Ara*f*]$_y$-[(1,4)-β-D-Gal*p*]$_z$; x, y, and z=1-3 (type I arabinogalactan motif)	epitope is masked by long galactan side chains	(1,4)-Gal, (1,5)-Ara	[31]
LM25	XLLG, XXLG and XXXG oligosaccharides of xyloglucan (X=xylose bound to G; G =glucose, L=galactose bound to xyloseX)	XLLG	XXXG and XXLG	[32]
LM21	β-(1,4)-Man, DP 2-5. Man_5, Gal_2Man_5	galactomannan-derived oligosaccharides	Man_4,Man_5	[33]
LM11	β-Xyl-(1,4)-β-Xyl-(1,4)-β-Xyl-(1,4)-β-Xyl	DP=3; glucuronoarabinoxylan (38% of Ara, 8% of GlcA)	β-1,4-xylan (DP>3), 4-O-methylglucuronoxylan (18% of MeGlcA), arabinoxylan (6% or 37% of Ara)	[34]
CBM3a	crystalline cellulose	xyloglucan	crystalline cellulose	[35,109]

References are numbered in accordance with the main text.

23. Manfield, I.W.; Bernal, A.J.; Moller, I.; et al. Re-Engineering of the PAM1 Phage Display Monoclonal Antibody to Produce a Soluble, Versatile Anti-Homogalacturonan scFv. *Plant Sci.* **2005**, *169*, 1090–1095. https://doi.org/10.1016/j.plantsci.2005.07.008.

24. Christiaens, S.; Van Buggenhout, S.; Ngouémazong, E.D.; Vandevenne, E.; Fraeye, I.; et al. Anti-Homogalacturonan Antibodies: A Way to Explore the Effect of Processing on Pectin in Fruits and Vegetables? *Food Res. Int.* **2011**, *44*, 225–234. https://doi.org/10.1016/j.foodres.2010.10.031

25. Ralet, M.C.; Tranquet, O.; Poulain, D.; Moise, A.; Guillon, F. Monoclonal Antibodies to Rhamnogalacturonan I Backbone. *Planta* **2010**, *231*, 1373–1383. https://doi.org/10.1007/s00425-010-1116-y.

27. Jones, L.; Seymour, G.B.; Knox, J.P. Localization of Pectic Galactan in Tomato Cell Walls Using a Monoclonal Antibody Specific to (1→4)-β-D-Galactan. *Plant Physiol.* **1997**, *113*,1405–1412. https://doi.org/10.1104/pp.113.4.1405.

28. Torode, T.A.; O'Neill, R.; Marcus, S.E.; et al. Branched Pectic Galactan in Phloem-Sieve-Element Cell Walls: Implications for Cell Mechanics. *Plant Physiol.* **2018**, *176*, 1547–1558. https://doi.org/10.1104/pp.17.01568.

108. Andersen, M.C.; Boos, I.; Marcus, S.E.; Kračun, S.K.; Rydahl, M.G.; Willats, W.G.; Knox, J.P.; Clausen, M.H. Characterization of the LM5 pectic galactan epitope with synthetic analogues of β-1,4-D-galactotetraose. *Carbohydr Res.* **2016**, *436*, 36–40. https://doi.org/10.1016/j.carres.2016.10.012.
29. Willats, W.G.T.; Marcus, S.E.; Knox, J.P. Generation of a Monoclonal Antibody Specific to (1→5)-α-L-Arabinan. *Carbohydr.Res.* 1998, *308*, 149–152. https://doi.org/10.1016/S0008-6215(98)00070-6.
30. Verhertbruggen, Y.; Marcus, S.E.; Haeger, A.; Verhoef, R.; Schols, H.A.; McCleary, B.V.; McKee, L.; Gilbert, H.J.; Knox, J.P. Developmental Complexity of Arabinan Polysaccharides and Their Processing in Plant Cell Walls. *Plant J.* **2009**, *59*,413–425. https://doi.org/10.1111/j.1365-313X.2009.03876.x.
31. Buffetto, F.; Cornuault, V.; Rydahl, M.G. The Deconstruction of Pectic Rhamnogalacturonan I Unmasks the Occurrence of a Novel Arabinogalactan Oligosaccharide Epitope. *Plant Cell Physiol.* **2015**, *56*, 2181–2196. https://doi.org/10.1093/pcp/pcv128.
32. Pedersen, H.L.; Fangel, J.U.; McCleary, B.L.; Ruzanski, C.; Rydahl, M.G.; Ralet, M.C.; Guillon, F.; Marcus, S.E.; Verhertbruggen, Y.; Knox, J.P.; et al. Versatile High-Resolution Oligosaccharide Microarrays for Plant Glycobiology and Cell Wall Research. *J. Biol. Chem.* **2012**, *287*, 39429–39438. https://doi.org/10.1074/jbc.M112.396598.
33. Marcus, S.E.; Blake, A.W.; Benians, T.A.; et al. Restricted Access of Proteins to Mannan Polysaccharides in Intact Plant Cell Walls. *Plant J.* **2010**, *64*,191–203. https://doi.org/10.1111/j.1365-313X.2010.04319.x.
34. McCartney, L.; Marcus, S.E.; Knox, J.P. Monoclonal Antibodies to Plant Cell Wall Xylans and Arabinoxylans. *J. Histochem. Cytochem.* **2005**, *53*, 543–546. https://doi.org/10.1369/jhc.4B6578.2005.
35. Blake, A.W.; McCartney, L.; Flint, J.E.; Bolam, D.N.; Boraston, A.B.; Gilbert, H.J.; Knox, J.P. Understanding the Biological Rationale for the Diversity of Cellulose-Directed Carbohydrate-Binding Modules in Prokaryotic Enzymes. *J. Biol. Chem.* **2006**, *281*, 29321–29329. https://doi.org/10.1074/jbc.M605903200.
109. Hernandez-Gomez, M.C.; Rydahl, M.G.; Rogowski, A.; Morland, C.; Cartmell, A.; Crouch, L.; Labourel, A.; Fontes, C.M.G.A.; Willats, W.G.T.; Gilbert, H.J. et al. Recognition of xyloglucan by the crystalline cellulose-binding site of a family 3a carbohydrate-binding module. *FEBS Lett.* **2015**, *589(18)*, 2297–2303. https://doi.org/10.1016/j.febslet.2015.07.009.
