# Supplementary material for: A Fresh Look at Celery Collenchyma and Parenchyma Cell Walls Through a Combination of Biochemical, Histochemical, and Transcriptomic Analyses
Source: Int J Mol Sci. 2025 Jan 16;26(2):738. doi: 10.3390/ijms26020738 (PMC11765706; doi:10.3390/ijms26020738)
Supplement: Supplementary file 1 [file ijms-26-00738-s001.zip › Table S3.pdf]

Table S3. GO Enrichment Results for genes upregulated in different clusters. For the analysis, only genes upregulated more than 2 or 4 times compared to other samples were selected (Table S2). The top 10 pathways and genes are presented; these genes are not corresponded to GO Enrichment results. The False Discovery Rate (FDR) cutoff 0.05; nG – the number of expressed genes in the cluster. The number of categories for some clusters was less than 10, since different numbers of genes were used for the GO enrichment analysis based on the criteria described in the table. Col – collenchyma, Par – parenchyma, Vas – vascular bundle.

Pathways	FDR	nG	Pathway Genes	Gene description (according to annotation for Arabidopsis	
**Cluster #1 (Par2/other tissues≥4)**					
Cell resp to hypoxia	5.60E-03	6	240	Ag8G00415	Senesc associated gene 20
Cell resp to decr oxygen levels	5.60E-03	6	242	Ag10G00411	Peroxisomal protein
Response to hypoxia	5.60E-03	6	267	Ag3G01863	Transcription factor HBP-1b
Response to oxygen	5.60E-03	6	272	Ag9G00088	Cysteine protease RD19B
Cell resp to chem stimulus	3.30E-02	13	1791	Ag11G03396	Transcription factor bHLH162
Response to chemical	1.40E-02	19	3091	Ag6G00194	Polygalacturonase
				Ag11G03404	ERF4
				Ag2G01515	UDP-glycosyltransferase 73B4
				Ag2G02593	PMEI13
				Ag9G00382	Peroxidase
**Cluster #2 (Col1/other tissues≥4)**					
Cutin biosynthetic proc.	3.20E-07	7	24	Ag5G02814	LTP
Plant-type cell wall organiz	4.00E-07	13	154	Ag5G00125	DUF642
Fatty acid metabolic proc.	2.80E-07	17	277	Ag7G01953	DUF642
Lipid catabolic proc.	3.30E-06	15	262	Ag5G00066	Expansin-A5
Cell wall organization	2.90E-09	26	532	Ag4G01175	XTH7
External encapsulating structure organization	1.70E-09	28	579	Ag9G01430	LRR
Polysacchar metabolic proc.	1.50E-06	21	495	Ag6G02015	DUF538
Cell wall organization or biogenesis	7.00E-09	29	700	AgUnG00730	GDSL esterase
Lipid metabolic proc.	2.60E-09	39	1161	Ag8G01854	MLP31
Anatom struct morphogenesis	1.90E-06	31	1043	Ag4G00902	Expansin-A15
**Cluster #3 (Vas1//other tissues≥4)**					
Nuclear chromos segregation	6.80E-09	16	121	Ag10G01218	LTPL101
Mitotic cell cycle proc.	6.50E-15	28	230	Ag7G01700	Aspartyl protease AED3
Mitotic cell cycle	1.50E-18	35	291	Ag4G00739	Protein sieve element occlus b
Nuclear division	3.90E-13	25	208	Ag3G02764	NRT1/ PTR family 6.2
Cell cycle proc.	2.10E-27	53	471	Ag4G02357	ENODL9
Organelle fission	1.70E-11	25	246	Ag2G02831	NRT1/PTR family 6.2
Microtubule-based proc.	3.40E-10	23	237	Ag1G01259	Protein sieve element occlus b
Cell cycle	2.50E-29	65	688	Ag8G01916	LTPL101
Reg. of cell cycle	2.70E-10	24	256	Ag11G04242	Histone H2A.1
Cell division	1.90E-18	42	450	Ag5G00724	Protein sieve element occlus b
**Cluster #4 (Par1/Par2; Col1/Col2; Vas1/Vas2≥2)**					
Protein oxidation	2.80E-02	2	5	Ag11G04333	Aquaporin TIP1-1
Peptidyl-cysteine oxidation	2.80E-02	2	5	Ag3G01713	Protein similar to pollen allerg
Detection of oxygen	3.40E-02	2	6	Ag4G02736	Annexin a1
Detection of hypoxia	3.40E-02	2	6	Ag4G00892	not classified

Pectin metabolic proc.	2.80E-02	6	167	Ag2G03025	L-ascorbate oxidase
Galacturonan metabolic proc.	2.80E-02	6	168	Ag2G00838	Pectin acetylesterase 8
Cell wall organization	7.20E-03	12	532	Ag1G01690	Arabinogalactan protein
Cell wall biogenesis	2.20E-03	15	700	Ag8G01384	Expansin-A15
External encapsulat struct organiz	8.20E-03	12	579	Ag10G02317	Isocitrate dehydrogenase
Carbohydrate metabolic proc.	7.90E-03	18	1183	Ag3G00314	FLA9
**Cluster #5 (Vas1,2/other tissues≥2)**					
Phloem or xylem histogenesis	5.90E-07	13	127	Ag1G00059	Sugar efflux transporter (SWEET7)
Regionalization	1.90E-06	13	172	Ag10G01002	Amine oxidase-related
Reg. of transcr DNA-templated	1.60E-06	54	2384	Ag1G00119	not classified
Reg. of nucl acid-templated transc	1.60E-06	54	2384	Ag1G01556	BRO1-like domain
Reg. of RNA biosynthetic.	1.60E-06	54	2384	Ag3G01241	Protein kinase domain
Reg. of nucleobase-contain comp	1.20E-06	59	2623	Ag3G02178	not classified
Transcription DNA-templated	1.90E-06	56	2560	Ag4G00397	Sulfurtransferase 18
Reg. of nitrogen comp metabol	1.30E-06	65	3099	Ag4G01475	Little zipper 3
Reg. of primary metabolic proc.	1.30E-06	66	3171	Ag4G00069	Protein kinase domain
Reg. of cellular metabolic proc.	1.90E-06	66	3295	Ag2G02299	Ferredoxin-fold anticodon-binding
**Cluster #6 (Par1/other tissues≥4)**					
Oxylipin biosynthetic proc.	5.70E-06	6	30	Ag7G02073	Zinc finger
Defense response	8.00E-09	40	1704	Ag2G01099	Cation exchanger 1
Response to extern biotic stimulus	2.60E-07	34	1485	Ag8G01743	MLP-like protein 423
Response to other organism	2.60E-07	34	1485	Ag11G04121	Asparagine synthetase
Biol proc. inv in interspecies interac	2.90E-07	34	1507	Ag6G00623	Vacuolar invertase
Response to external stimulus	8.00E-08	41	1947	Ag1G00642	Cysteine-rich secretory proteins
Cell response to chemical stimulus	1.50E-06	36	1791	Ag4G02737	LRR-XII protein kinase
Response to stress	5.80E-09	66	3953	Ag10G00253	LTPL113
Response to chemical	4.60E-06	49	3091	Ag6G01954	Chitinase A
				Ag3G01904	not classified
**Cluster #7 (Vas2/other tissues≥2)**					
Transition metal ion homeostas	2.00E-06	12	110	Ag3G00376	Metallothionein-like 3B
Metal ion homeostasis	2.00E-06	15	185	Ag4G01511	Phloem 2-like A5
Chemical homeostasis	1.90E-06	23	433	Ag4G01510	Phloem 2-like A5
Transmembrane transport	1.90E-12	55	1301	Ag2G02209	WALLS ARE THIN 1
System development	2.00E-06	57	2070	Ag10G02702	Glycine-rich protein
Reg. of transcrip DNA-templated	2.00E-06	62	2384	Ag4G02274	Jacalin-related lectin 3
Transport	1.90E-06	68	2669	Ag4G00003	Sugar efflux transporter (SWEET)
Establishment of localization	1.90E-06	69	2715	Ag5G02115	not classified
Response to chemical	4.10E-07	78	3091	Ag4G02201	Asparaginase B1
Anatomical structure develop	2.00E-06	75	3130	Ag6G02337	MYB48
**Cluster #8 (Par1,2/other tissues≥2)**					
Acidic amino acid transport	3.10E-03	3	10	Ag7G00208	Aspartyl protease

Protein phosphorylation	8.60E-07	30	1182	Ag11G04650	Gibberellin receptor GID1L2
Response to extern biotic stimul	1.10E-04	29	1485	AgUnG01092	Dehydrogenase
Response to other organism	1.10E-04	29	1485	Ag6G02719	Exordium like 2
Biol proc. involv in interspec inter	1.10E-04	29	1507	Ag3G02525	GASR6 - Gibberellin-regulated
Phosphorylation	1.10E-04	30	1591	Ag2G01464	Delta(12)-fatty-acid desaturase
Defense response	3.10E-04	30	1704	Ag3G00095	Amino acid permease 6
Response to external stimulus	2.40E-04	33	1947	Ag6G00226	WRKY15
Response to stress	1.60E-03	50	3953	Ag4G02899	Heavy metal transport
				Ag4G00542	not classified
**Cluster #9 (Par2/Par1, Col2/Col1, Vas2/Vas1≥2)**					
Response to light stimulus	1.20E-03	23	773	Ag8G01639	Aluminum induced protein
Response to radiation	1.40E-03	23	796	Ag3G02497	Responsive to dehydration 19
Hormone-mediated signaling	3.70E-03	25	1014	Ag7G02138	Ankyrin repeat
Cell response to organic substance	1.70E-03	30	1263	Ag9G02614	α-vacuolar proces enzyme
Response to abiotic stimul	5.70E-06	51	2170	Ag2G00877	E3 ubiquitin ligase (XBAT3)
Cell response to chem stimulus	2.50E-03	37	1791	Ag4G02495	ABC transporter G
Response to endogenous stimul	5.00E-03	34	1669	Ag1G01900	Aluminum induced protein
Response to organic substance	2.00E-03	41	2051	Ag4G02847	Subtilisin-like protease SBT1.7
Response to chemical	1.20E-03	57	3091	Ag1G00960	Glucose-1-P adenylyltransferase
Cellular response to stimulus	1.70E-03	58	3315	Ag6G02891	Aspartic proteinase A1
**Cluster #10 (Col1,2/other tissues≥1.5)**					
Hormone-mediated signaling	9.50E-03	23	1014	Ag5G00400	XTH6
Cell response to hormone stimul	9.50E-03	23	1076	Ag9G02472	Phospholipase-A2 (pPLA2-III))
Cell response to endogen stimul	9.50E-03	23	1107	Ag4G00316	TORTIFOLIA1-like 4
Cell response to organic substance	1.40E-02	24	1263	Ag8G00125	RRT9
Response to hormone	9.50E-03	30	1639	Ag6G00595	Microtubule-associated 70-1
Response to endogenous stimulus	9.50E-03	30	1669	Ag5G01163	PME17
Signal transduction	1.20E-02	33	1986	Ag7G00500	Phospholipase-A2 (pPLA2-III))
Response to organic substance	1.20E-02	34	2051	Ag9G00597	Kinesin light chain-related 2
Signaling	1.40E-02	33	2029	Ag1G01801	Microtubule-stabilizing factor (WDL)
Response to chemical	1.70E-02	44	3091	Ag3G01636	Glutamine synthetase isozyme
